# Determinants of the incidence of non-academic staff in European and US HEIs

**DOI:** 10.1007/s10734-022-00819-7

**Published:** 2022-02-17

**Authors:** Alessandro Avenali, Cinzia Daraio, Joanna Wolszczak-Derlacz

**Affiliations:** 1 Dipartimento Di Ingegneria Informatica Automatica E Gestionale Antonio Ruberti (DIAG), Sapienza University of Rome, Rome, Italy; 2 Faculty of Management and Economics, Gdańsk University of Technology, Narutowicza 11/12, 80-233 Gdańsk, Poland

**Keywords:** Higher education institutions, Proportion of non-academic staff, Determinants of non-academic staff, Europe, USA, I22, I23, C14

## Abstract

In this article, we contribute to the scant literature covering quantitative studies on the determinants of the non-academic staff incidence in higher education institutions by analysing how the proportion of non-academic staff is related to key features such as size, prestige, year of foundation and financial structure of universities. We apply nonlinear regression analysis to compare HEIs across Europe and the USA, taking into account time and cross-country heterogeneity of the two balanced panel datasets concerning European and American universities over a period of 6 years (2011–2016 for Europe and 2012–2017 for the USA). Evidence suggests that in both Europe and the USA, public and larger (if sufficiently large) as well as more research-oriented units are characterised by a higher proportion of non-academic staff. In Europe, we observe an inverted U-shaped effect of the share of non-personnel expenditure and the foundation year on the proportion of non-academic staff, while the proportion of non-academic staff decreases with the share of core and third-party funding. For the USA, we obtain similar findings except that the share of core funding and third-party funding is characterised by a U-shaped effect, and the impact of the share of non-personnel expenditure has no empirical effect on the proportion of non-academic staff. Additionally, we discover that some factors that contribute to the proportion of non-academic staff may constitute indicators of performance, suggesting the need for further research to extend our knowledge on the complex issue of the role played by non-academic staff in university performance.

**Supplementary Information:**

The online version contains supplementary material available at 10.1007/s10734-022-00819-7.

## Introduction

A key factor determining the success of any organisation is its staff. According to the tasks they perform, employees of higher education institutions (HEIs) can be divided into several organisational domains: researchers and teachers (the main executives of university primary processes), administrative and technical staff (in charge of organising and supporting primary processes) and high professional administrators (who coordinate and organise the activities of employees of the other domains). However, quantitative data presented in the relevant literature often oversimplify personnel division by applying a binary model where total staff is divided into academic staff (researchers and teachers) and non-academic staff (all other employees different from researchers and teachers).^1^

While the role of academics in university performance is usually underlined and explored in-depth, the impact of non-academic staff on academics’ outcomes related to institutional outputs cannot be neglected. Unfortunately, recent investigations and assessments of the activities and roles of non-academic staff in the functioning of universities are not very favourable. Ginsberg (2011) dedicates an entire book to describe ongoing and disturbing changes in the operation of universities, with an increasingly higher impact of (senior and middle) professional administrators’ decisions on the rules and the priorities of academic life.^2^ Next, as one of the main problems concerning the functioning of today’s universities, Martin (2016) points to the growth of bureaucratic procedures and, as a result, a disproportionate increase in the number of employees holding administrative positions. However, despite the growth in non-academic staff, academics employed by universities spend more and more time on non-academic (bureaucratic) activities (Kwiek, 2018).

Surprisingly, there is a scarcity of literature devoted to developing quantitative studies aimed at investigating key factors affecting the size of non-academic staff (i.e. the number and the share of non-academic personnel employed by HEIs) or finding empirical evidence concerning the role of non-academic staff in university performance. In an empirical analysis of universities in 11 European countries for the 2011/2012 academic year, Baltaru and Soysal (2018) find that the main factor influencing the growth of administration is the engagement of HEIs in multiple activities and undertaking various (new) missions and initiatives. Indeed, the proliferation of non-academic staff is mainly the result of transformation of universities into organisations that pursue strategic missions related both to primary tasks assigned to universities such as teaching, research and third mission activities and to public-policy goals such as inclusion and equality issues.^3^ Furthermore, a recent study concerning the sample of 100 British universities conducted by Baltaru (2019a) examines whether changes in the staffing structure between 2003 and 2011 affected the performance of the universities. She finds that universities that moderately increased the number of non-academic personnel were characterised by higher rates of course completion, but finds no such effect on research quality, good honours degrees or graduate employability. Baltaru (2019a) concludes that university performance is solely determined by the reputation and prestige of the institution.

## Aim and contribution

The main purpose of this paper is to shed new light on the main factors that determine the incidence of non-academic staff in higher education institutions (HEIs). We contribute to the scant existing literature covering quantitative studies on the determinants of the non-academic staff incidence in higher education institutions by developing an empirical analysis, based on the estimation of nonlinear regressions, to compare the employment structure (ratio of non-academic staff to total staff) of European HEIs and their US counterparts. Two unique databases are used for the analysis. For Europe, we merge individual information on HEIs from the European Tertiary Education Register (ETER) with bibliometric indicators from the Centre for Science and Technology Studies (CWTS) at Leiden University.^4^ For the USA, the main data source is the Integrated Postsecondary Education Data System (IPEDS) merged with publication data from the CWTS and information on the year of foundation obtained directly from the websites of institutions.

Our objective is to extend pioneering cross-sectional results on determining factors concerning non-academic staff in European HEIs explored by Baltaru and Soysal (2018) for the 2011/2012 academic year by analysing the dynamics over time in European HEIs, including the most updated data available for Europe, and providing an *indirect* comparison between European and US HEIs by taking into account time and cross-country heterogeneity. This means that we will analyse the two systems (European and American) separately but use the most similar variables available for each system in our elaborations. To the best of our knowledge, this is the first study to focus on the comparison concerning the incidence of non-academic staff in HEIs. On the other hand, Wolszczak-Derlacz (2017) provides a comparison of the efficiency of the two systems, while Lepori et al. (2019) analyse European and American scientific production.

Comparing Europe with the USA is useful and interesting for several reasons. First of all, many European countries are implementing reforms of their higher education systems based on the American model even if there is a lack of quantitative and systematic analyses to understand which elements of the American system are considered most suitable for the European context and which ones should be disregarded (see also Aghion et al., 2010). Second, the preliminary step for conducting the comparison is the collection and analysis of existing data on the two systems. As a result, carrying out the comparison enables us to thoroughly investigate the availability and consistency of data and provides us with useful suggestions on which data could be improved or integrated in future data collections. As we will see, the US data available on non-academic staff are much more detailed than the European data included in ETER. Finally, an indirect comparison, as the one carried out in this paper, can provide an overall and updated view on the incidence of non-academic staff in the two systems while keeping track of their heterogeneity.

The rest of the paper is structured as follows. The “Relevant literature” section includes a summary of the previous studies, taking into account universities’ features that may affect the incidence of non-academic staff. In the “Data and descriptive analysis” section, we describe two panel datasets, along with key descriptive statistics on the European and American HEIs. The “Methods” section illustrates the nonlinear regression approach followed in the empirical analysis to model the nonlinear relationship that we find between the share of non-academic staff (the dependent variable) and its potential determinants. The “Results” section includes the main results, while the “Discussion and further developments” section focuses on the discussion of the findings and the outline of further research. The “Concluding remarks” section contains the concluding remarks.

## Relevant literature

The long-observed increase in the number of administrative and technical personnel in higher education institutions is a universal trend that emerged in many countries around the world (Grove, 2012; Gumport & Pusser, 1995; Hansen & Guidugli, 1990; Krücken, 2011; Krücken et al., 2013; Rhoades & Sporn, 2002; Sebalj et al., 2012). Nowadays, non-academic staff usually comprises a large part of the total staff of universities and, in many cases, is even larger than academic staff. Indeed, new and complex external and internal challenges in the last decades caused administrative and technical activities within HEIs to increase considerably and evolve accordingly. However, most of these activities were allocated to administrative or technical personnel of universities (usually referred to as “general staff” or “non-academic staff”), while several of them were outsourced or left under the charge of academic staff.

As a consequence, the last decades saw a wide development of general staff within universities in terms of its growth, higher responsibilities and more crucial roles. General staff contributes to the definition of strategic targets and internal rules and to the overall performance (e.g. by collaborating with academic staff on complex projects, providing technological support to teaching and student learning). Therefore, non-academic staff is usually located in every organisational structure of the university (e.g. departments, faculties, central offices). It is also typically characterised by a multitude of very different professional roles. For instance, general staff may include technical personnel, maintenance staff, office workers and high professional administrative personnel^5^ (for the discussion on the general staff evolution, see, e.g. Szekeres, 2004, 2011; Whitchurch, 2006, 2007, 2017; Sebalj et al., 2012; Kallenberg, 2018; Smith et al., 2021).

Despite the involvement of general staff in many different skills and roles, quantitative models and data presented in the relevant literature are unable to represent the complexity underlying non-academic staff for many HEIs considered in our analysis. In particular, the binary staff classification (academic/non-academic) does not provide the full picture of the complexity of personnel classification within HEIs (Sebalj et al., 2012; Smith et al., 2021; Szekeres, 2011; Whitchurch, 2006, 2007, 2013). In this study, however, we will still refer to it because most of the available quantitative staff data are provided in binary format. The identification of key factors that may play a role in affecting the level of the overall non-academic personnel inside universities is an interesting research issue, which can be investigated by applying binary staff data.

Some first crucial reasons may be explained by taking inspiration from the long-term goals of any HEI. Indeed, to deal with the strong domestic and international competition in terms of attracting more students and external funding for large research projects (above all, due to a shortage of core funding such as government grants), universities must strengthen their non-academic structures with highly professional administrative personnel who can take part in the strategic planning process (Baltaru, 2019a; Gornitzka & Larsen, 2004; Graham, 2013; Kallenberg, 2018; Mcinnis, 1998; Ramirez & Christensen, 2013; Tolofari, 2005; Veles & Carter, 2016). Therefore, a higher number of students and larger external funding could trigger an increase in non-academic staff. The management of a growing number of students and complex research projects also poses a difficult challenge in terms of efficiency, possibly requiring an administrative structure that includes larger staff with many different skills (Baltaru & Soysal, 2018; Gibb et al., 2012).^6^

In recent decades, the new public management paradigm also led universities to change their organisation and governance in such a way to conduct their operations in a similar fashion to private organisations, where an effective and sufficiently large administrative structure, supporting primary processes, is crucial to ensure that an organisation is successful (Baltaru & Soysal, 2018; Bleiklie et al., 2011; De Boer et al., 2007; Deem et al., 2007; Pollitt & Bouckaert, 2004; Szekeres, 2004, 2006, 2011; Tolofari, 2005). This led to a relationship between academics and general staff, which is often described as conflicting and characterised by scarce understanding (Anderson, 2008).^7^ This corporatisation process of universities can be usually observed in countries where public education systems are predominant. Thus, the country of origin too might affect the general staff increase observed in last decades.

Furthermore, important worldwide rankings unavoidably provide universities with suitable incentives to control and, if necessary, modify their organisational behaviour in order to improve their position in such rankings. An effective management control system requires non-academic staff to be empowered in terms of their number and competences (Bromley & Meyer, 2014; Sauder & Espeland, 2009).

It is worth noting that the increase in the number of non-academic staff is in some cases related to a quite large increment of highly qualified administrative personnel and to a decrement of technical and clerical staff, most likely due to the outsourcing of non-core activities (Gornitzka & Larsen, 2004; Whitchurch, 2006, 2007). With this in mind, increased expenditures on tenders of services may be associated with a decrement of technical and clerical personnel.

Still, most of the studies included in the relevant literature are based on qualitative analyses and fail to investigate whether geographical and institutional (Europe vs. USA) features may have an impact on the non-academic staff expansion. A notable exception is the paper by Baltaru and Soysal (2018), where the authors apply a multivariate linear regression model to study factors associated with the variation in the percentage of non-academic staff across European universities for the 2011/2012 academic year. However, they still fail to consider the potential role of different geographical and institutional contexts.

## Data and descriptive analysis

As far as European HEIs are considered, the main source of data is the European Tertiary Education Register (ETER, www.eter-project.com), which provides open access to a cross-country database at the level of individual HEIs, containing information about their characteristics such as financial resources, staff, student enrolment and graduates. Currently (as at June 2021), the data are available for 3,280 HEIs from 37 countries and for academic years from 2011 to 2016.^8^ The establishment of one, common and publicly available database of HEIs in Europe such as ETER is a milestone for data collection. However, the level of data completeness significantly varies between countries, domains and variables. In this paper, we merge data on HEIs characteristics from ETER with bibliometric information (based on the number of publications of academics affiliated with a given institution indexed in the Web of Science) from the Centre for Science and Technology Studies (CWTS) at Leiden University.^9^

Our analysis is restricted to the sample of universities defined as academic institutions with the right to award doctoral degree (as opposed to university of applied sciences, colleges, vocational schools such as Fachhochschule in Austria and Germany, Hogescholen in the Netherlands, colleges in Norway, Szkoły Zawodowe in Poland).^10^ Hence, specialised units such as arts/music/sport/police and theological academies are not taken into account. Additionally, distance education universities are excluded where off-campus teaching (e.g. through online courses) constitutes a substantial component of the educational offer, affecting the staff structure and student-faculty ratios.^11^ Furthermore, the sample is limited to the balanced panel of those HEIs reported for the subsequent 6 years—from 2011 to 2016—and with the minimum number of 500 students to avoid the inclusion of smaller outliers in the analysis. It is very often the case that some data recorded in ETER are not complete. Also, for some countries, the number of academic and non-academic staff is reported in FTE (full-time equivalent) and for others in HC (head count). To overcome these problems, basic variables (if missing) were imputed.^12^ We calculate the ratio of non-academic to total staff (*Non_acad)* and treat FTE as the basic classification.^13^

**Fig. 1:**
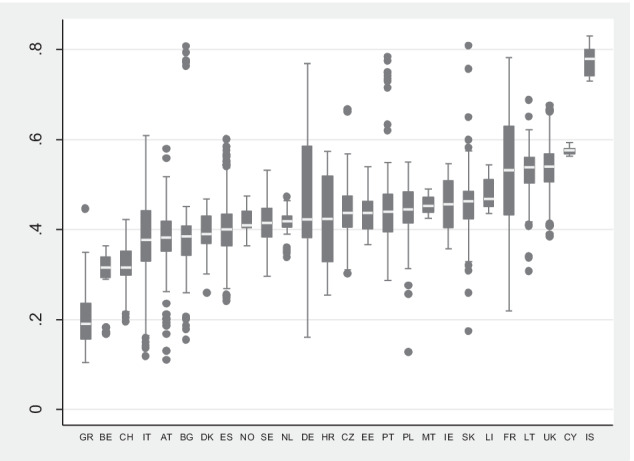
Proportion of non-academic staff to total staff across different countries (all years pooled together). Source: authors’ own elaboration based on ETER data

In the Appendix, in Table 4, the European coverage of our final sample after an initial outliers’ detection is presented.^14^ As a result, information on 675 HEIs from 26 countries were obtained. In Fig. 1, cross-country differences of the proportion of non-academic staff to total staff are presented. The mean value is 45%, with Iceland, Cyprus and the UK being the countries with the highest share of non-academic staff to total staff, whereas Greece, Belgium and Switzerland with the lowest. For larger countries in which the number of reported HEIs is relatively high, the dispersion is substantial; for example, in Italy, there are institutions where non-academic staff constitutes less than 20% of total staff, as well as institutions with the majority of non-academic staff, i.e. 60% of total staff, similarly in Germany and the UK. Interestingly, the mean share of non-academic staff in total staff is stable over time (see Table 5 in the Appendix).

The main source of data for higher education institutions from the USA is the Integrated Postsecondary Education Data System (IPEDS). The IPEDS covers all higher education institutions in the USA. In 2017, for instance, data were reported for 7,153 institutions. The IPEDS contains very detailed information on students, staff, salary, finance, etc. and it was merged with CWTS data on publication records as in the case of European HEIs. The final sample includes institutions classified as public or private not-for-profit 4-year or longer, including institutions conducting research, excluding specialist (one-field) institutions such as theological seminaries or medical schools (according to the Carnegie Classification), for which the CWTS provides data on publication records and which are recorded in all analysed years (balanced panel). The period of analysis is limited to years 2012–2017, which overlaps with the data for Europe. In the final sample of 341 institutions, there are 215 HEIs classified as public and 126 as private not-for-profit. The sample does not include private for-profit institutions whose financial structure differs significantly from those analysed.

We calculate the ratio of non-academic staff to total staff (*Non_acad*) which is expressed in FTE.^15^ For the USA, there is more detailed information available compared to Europe.^16^ The group of variables on non-academic staff is in fact detailed in the following occupational categories: librarians, management, business and financial operations, IT (Information Technologies), engineering and science; community, social service, legal, arts, design; entertainment, sports and media; healthcare practitioners and technical; service occupations; sales and related occupations; office and administrative support; natural resources, construction and maintenance; production, transportation and material moving. The employment structure in American HEIs is presented in Table 1. Overall, the average share of non-academic staff across American universities is around 64%, while office and administrative support constitutes the highest share of non-academic staff, which is 22% on average. Similar to European institutions, the share of non-academic staff to total staff is constant over time.

**Table 1:** Employment structure of the US HEIs

Variable	Obs	Mean	Std. Dev	Min	Max
**Non-academic staff to total staff (*Non_acad*)**	2,046	0.639	0.07	0.343	0.856
Share of a given non-academic occupational category in total non-academic staff, FTE					
Librarians, archivists, curators and museum/student and academic affairs	2,046	0.084	0.061	0.000	0.469
Management	2,046	0.142	0.079	0.000	0.567
Business and financial operations	2,046	0.113	0.071	0.000	0.674
IT, Engineering and Science	2,046	0.127	0.067	0.000	0.479
Community, social service, legal, arts, design, entertainment, sports and media	2,046	0.093	0.054	0.000	0.482
Healthcare practitioners and technical	2,046	0.037	0.056	0.000	0.501
Service occupations	2,046	0.125	0.053	0.000	0.308
Sales and related occupations	2,046	0.003	0.01	0.000	0.204
Office and administrative support	2,046	0.219	0.079	0.012	0.861
Natural resources, construction and maintenance	2,046	0.045	0.029	0.000	0.3
Production, transportation and material moving	2,046	0.012	0.012	0.000	0.094

Source: authors’ own elaboration based on IPEDS.

Table 2 includes basic descriptive statistics concerning our sample of European and US HEIs: the number of students, the number of students per FTE academic staff, the share of core revenues,^17^ the share of third-party revenues,^18^ the share of non-personnel expenditure,^19^ publications per academic staff member, proportion of publications belonging to top 10% most cited in the field and year, the average number of citations (impact) per publications (normalised by year and field).

**Table 2:** Descriptive statistics, yearly averages

Variable	Obs	Mean	Std. Dev	Min	Max
	Europe				
Students total	4050	16,787	13,149	525	112,472
Students per academic staff	4050	17.43	9.57	1.18	99.40
Non-personnel expenditure in total	2742	0.33	0.10	0.05	0.78
Core budget in total	2902	0.56	0.25	0.00	1.00
Third-party budget in total	2710	0.17	0.13	0.00	0.96
Publications per academic staff	4050	0.62	0.55	0.00	7.06
Top 10% publications	4050	0.11	0.05	0.00	0.50
Citation	4050	1.15	0.51	0.00	11.78
	USA				
Students total	2046	21,623	14,636	1,529	112,984
Students per academic staff	2046	20.05	8.99	0.78	74.39
Non-personnel expenditure in total	2042	0.53	0.05	0.31	0.83
Core budget in total	2042	0.22	0.20	0.00	0.92
Third-party budget in total	2042	0.15	0.11	0.00	0.58
Publications per academic staff	2046	0.69	0.62	0.00	7.48
Top 10% publications	2046	0.13	0.05	0.00	0.50
Citation	2046	1.30	0.49	0.00	10.99

Notes: Descriptive statistics refer to yearly averages, calculated on the sample of 675 universities in Europe (2011–2016) and 341 in the USA (2012–2017).

Source: authors’ own elaboration based on ETER and IPEDS.

Total revenues of HEIs are composed of two basic categories: core and third-party revenues. The classification of a given type of revenue into core and third-party funding is based on ETER and IPEDS documentation. For European universities, in ETER, core funding includes basic government allocations, gifts and donations, interests and investment income. For US universities, to ensure some comparability with European universities, the following IPEDS items were included in core funding: federal, state and local appropriations, federal, state and local non-operating grants, gifts, other revenues and investments. For European universities, third-party funding includes public and private grants and contracts and grants and contracts from abroad, while for US universities, it covers federal, state and local grants and private gifts, grants and contracts. This correspondence between European and US revenues as divided into core and third-party categories, although not perfect, proved to be effective also in other studies such as Lepori et al. (2019), from which it is derived.^20^

On average, the US institutions are larger (the average number of students in the US HEIs and the European HEIs is 21,600 and approx. 17,000, respectively), while the largest units are similar in size as far as the number of students is concerned. The average number of students per academic staff is greater in the USA (20 students versus 17 students per academic staff). As far as the structure of finance, revenue sources and expenditure allocation are concerned, there are some differences between the European and American universities. Firstly, non-personnel expenditure as a share of total expenditure is higher in the US units (53% on average), while the average share of core budget in the total budget is lower in the US units, with the average share of third-party budget being equal to 17% and 15% for Europe and the USA, respectively. The indications of research outcomes measured by bibliometric records are similar for the European and American samples: 0.62 to 0.68 publications per academic staff (yearly), 11 to 13% share of top publications and citations from 1.15 to 1.3 comparing the average of European indicators to the American ones.

## Methods

We apply regression analysis to examine determinants of the share of non-academic staff in HEIs. Independent variables (potential determinants of the share of non-academic staff) are mostly selected based on the theoretical discussion in the “Relevant literature” section. In particular, the number of students and the share of third-party revenues may be considered significant potential explanatory variables since attracting a higher number of students and larger external funds for complex research projects is typical strategic goals of any HEI, which thus require to be effectively supported by general staff (Baltaru, 2019a; Baltaru & Soysal, 2018; Gibb et al., 2012; Gornitzka & Larsen, 2004). The share of core revenues may be considered an interesting variable to be considered as well. Indeed, on the one hand, larger core revenues for institutional activities could increase the total effort for non-academic staff and, consequently, the size of overall non-academic staff (Bromley & Meyer, 2014; Ginsberg, 2011; Graham, 2013). On the other hand, a lower level of core revenues could demand a larger efficiency of administrative staff (Bleiklie et al., 2011; Tolofari, 2005). With this in mind, in terms of the share of non-academic staff, the overall relationship with core revenues could be not univocally determined and could, most likely, be nonlinear. In addition, several worldwide rankings push universities to improve their outcomes such as, in particular, publications and graduate students, which, in turn, may require non-academic staff to be empowered in order to support and control the pursuit of these challenges (Sauder & Espeland, 2009). Therefore, the number of publications per academic staff member (considered a proxy of university’s scientific production and contribution to university’ rankings and prestige) is also expected to be an important determinant (in this paper, emphasis is not put on graduate students as they are correlated with the number of students). In some cases, several non-core activities supporting primary processes can be outsourced, the goal of which is to save money (mostly when the scale of these activities is large) and to obtain operational efficiency (Gornitzka & Larsen, 2004; Whitchurch, 2006, 2007). The share of non-personnel expenditure could partially^21^ capture this aspect, showing some negative impact on the incidence of non-academic staff. Furthermore, the corporatisation process of many HEIs (due to the spread of the new public management paradigm) generally extended the role and responsibilities of general staff, while its implementation can be dependent on the specific country and be observed mostly in areas characterised by a prevailing incidence of public HEIs (De Boer et al., 2007; Deem et al., 2007; Pollitt & Bouckaert, 2004; Szekeres, 2006; Tolofari, 2005). The country of origin and the public/private nature of the HEI could, therefore, help capturing the impact of this organisation and governance change on the incidence of non-academic staff. Finally, the foundation year and time fixed effects are considered to be further potential explanatory variables related to the age of the HEI and to the role of time, respectively.

Unlike previous studies, which use linear regressions (Baltaru & Soysal, 2018), we carried out nonparametric analysis before defining our model. It was based on locally weighted regressions (*lowess*) between some potential explanatory variables and the share of non-academic staff (dependent variable). The objective of this preliminary nonparametric analysis is to identify the relationship between the dependent variable and the explanatory variables without assuming it a priori. Another aspect that makes our analysis a novel contribution to the field is that the elaborations are based on data on individual HEIs from several years (2011/2012–2016/2017).

**Fig. 2:**
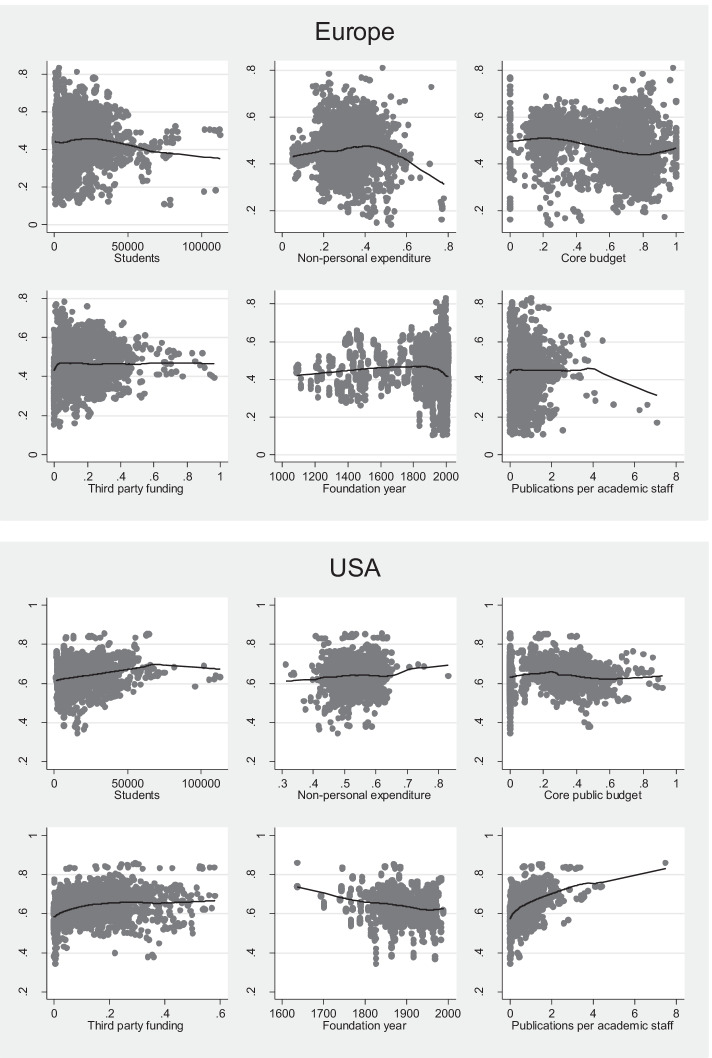
Proportion of non-academic staff to total staff (on the y-axes) versus the analysed variables (the number of students, non-personnel expenditure, core budget, third-party funding, foundation year, publications per academic staff). Upper panel, Europe; lower panel, USA. Source: authors’ own elaboration based on ETER and IPEDS

Figure 2 illustrates *lowess* scatterplots separately for the European sample (the upper panel) and the US sample (the lower panel). Following the detection of evidence of nonlinear relationships that can be seen in Fig. 2, a *nonlinear regression model* is developed in order to analyse the determinants of the share of non-academic staff for each dataset available. Conducting two separate analyses allows us to make an *indirect* comparison useful for keeping track of the heterogeneity of the datasets^22^ and, at the same time, makes it easier to carry out a comparative evaluation of the results for the two systems.

Generally, nonlinear trends are observed for both the European and the American sample. The first analysed variable is the number of students to see if larger institutions are characterised by a higher share of non-academic staff. For Europe and the US alike, an increasing trend can be observed up to the largest institutions where the trend is reversed. The next sub-graphs present the relationship between the share of non-personnel expenditure to total expenditure and the share of non-academic staff. The share of non-personnel expenditure can be considered a proxy of outsourcing activities of universities. In the case of the European institutions, the relationship appears to be nonlinear—the higher the non-personnel expenditure, the higher the share of non-academic staff up to a threshold above which the trend is reversed. For the US HEIs, the graph shows a rather flat line. Furthermore, a similar approach is used to analyse the relationship between the share of core funding and the share of third-party funding. Core funding refers to the funds available for the operations of the institution as a whole; usually, it is not earmarked for specific activities. In both cases, there are some nonlinear relationships against the share of non-academic staff. On the other hand, third-party funding appears to be deterministic to the share of non-academic staff only for the US HEIs.^23^ The relationship between the year of foundation and the share of non-academic staff (*Non_acad*) and the relationship between the publication per academic staff member and *Non_acad* are also presented. Publications per academic staff are considered a proxy of research orientation of the unit and prestige of the institution.

Using *lowess* smoothing-graph analyses pointing to certain nonlinear relationships between the potential explanatory variables and *Non_acad* (our dependent variable), we estimate of a *polynomial* function based on the variables proposed in previous studies, in particular by Baltaru and Soysal (2018), and on the relevant variables that were available in our databases.

The following model is proposed:

1 $${Non\_acad}_{ict}=\alpha +{{\beta }_{1}{\mathrm{Stud}}_{it}+{\beta }_{2}{\left({Stud}_{it}\right)}^{2}+{\beta }_{3}{YearFound}_{i}{+{\beta }_{4}{({YearFound}_{i})}^{2}+{\beta }_{5}{Private}_{i}+\beta }_{6}{Publ\_Acad}_{it}+\beta }_{7}{Non\_personal}_{it}+{{{\beta }_{8}(Non\_personal}_{it})}^{2}+{\beta }_{9}{Core\_budget}_{it}+{\beta }_{10}({{Core\_budget}_{it})}^{2}+{\beta }_{11}{Third\_party}_{it}+{{{\beta }_{12}(Third\_party}_{it})}^{2}{+D}_{c}+{D}_{t}+{\epsilon }_{ict}$$

where *i* is the individual university, *c* is either the country or state in the case of the American sample and *t* is the time. The dependent variable is the share of non-academic staff to total staff (*Non_acad)*, while the independent variables include the number of students enrolled (*Stud*), the foundation year of an institution (*YearFound*), indication whether the institution is a private one (*Private*), the number of publications per academic staff (*Publ_Acad*), the share of non-personnel expenditure to total expenditure (*Non_personal*), the share of core revenues (*Core_budget*) and the share of third-party revenues (*Third_party*) to their totals.

The data available refer to a short panel that covers 6 years of observations, meaning that we can only estimate a pooled model with country (or state) and time effects.^24^ More specifically, the independent variables include both time-variant and time-invariant regressors; the latter include the foundation year and type of institution (private versus pubic). For this reason, we cannot use a fixed-effects estimation method; otherwise, the time-invariant regressors would be dropped. Hence, in this paper, we consider individual effects for time (in order to gauge time-specific trends) represented by variable *D*_t_ and country (or state in the US sample case) effects represented by variable *D*_c_ in order to capture country/state specific characteristics (e.g. institutional factors of higher education systems). With this modelling strategy, our model can include all the variables in which we are interested. In addition, we employ a robust variance estimator in order to take into account the possible presence of heteroscedasticity. Finally, we calculate partial correlations among the independent variables to find if the regressors are independent among them—as they should be—in order to avoid the problem of *multicollinearity* that originates from correlated covariates and can affect the estimate reliability. Tables 6 and 7 in the Appendix include the partial correlations calculated for the European and US samples, respectively.^25^

Considering data heterogeneity and to check if the European and American institutions differ in relation to the determinants of *Non_acad*, we run Eq. 1 separately for these two groups of institutions.

## Results

The results of the nonlinear regression analyses carried out in relation to the European and American samples are presented in Table 3. Here, the results of the final regression with all covariates are given, while the Appendix (Tables 8 and 9) includes different versions of the specification, starting from a slimmer regression (with fewer right-hand variables and without financial variables) separately for the European and US HEIs. It should be noted that when additional variables are added, the number of observations in the European sample decreases, with the final specification being then estimated for HEIs from 20 (out of 26) European countries.

**Table 3:** Determinants of *Non_acad* (dependent variable: ratio of non-academic staff to total staff), European and US samples

	Europe	USA
	(1)	(2)
Students_it_	− 0.008***	− 0.004
	[0.003]	[0.003]
Students_it_^2^	0.001***	0.001**
	[0.000]	[0.000]
YearFound_i_	0.067***	0.356***
	[0.012]	[0.095]
YearFound_i_^2^	− 0.002***	− 0.010***
	[0.000]	[0.003]
Private_i_	− 0.028**	− 0.035***
	[0.014]	[0.010]
Publ_Acad_it_	0.024***	0.052***
	[0.004]	[0.004]
Non_personal_it_	0.922***	− 0.39
	[0.136]	[0.412]
Non_personal_it_^2^	− 1.243***	0.343
	[0.184]	[0.390]
Core_budget_it_	− 0.100**	− 0.173***
	[0.047]	[0.048]
Core_﻿budget_it_^2^	0.06	0.177***
	[0.047]	[0.058]
Third_ ﻿party_it_	− 0.111***	0.177***
	[0.037]	[0.045]
Third_party_it_^2^	0.088	− 0.489***
	[0.057]	[0.101]
*N*	2570	2042
*R*^2^	0.46	0.43

*p < 0.10, **p < 0.05, ***p < 0.01, country (European sample)/state (US sample) and time fixed effects included (not reported). Robust standard errors.

**Fig. 3:**
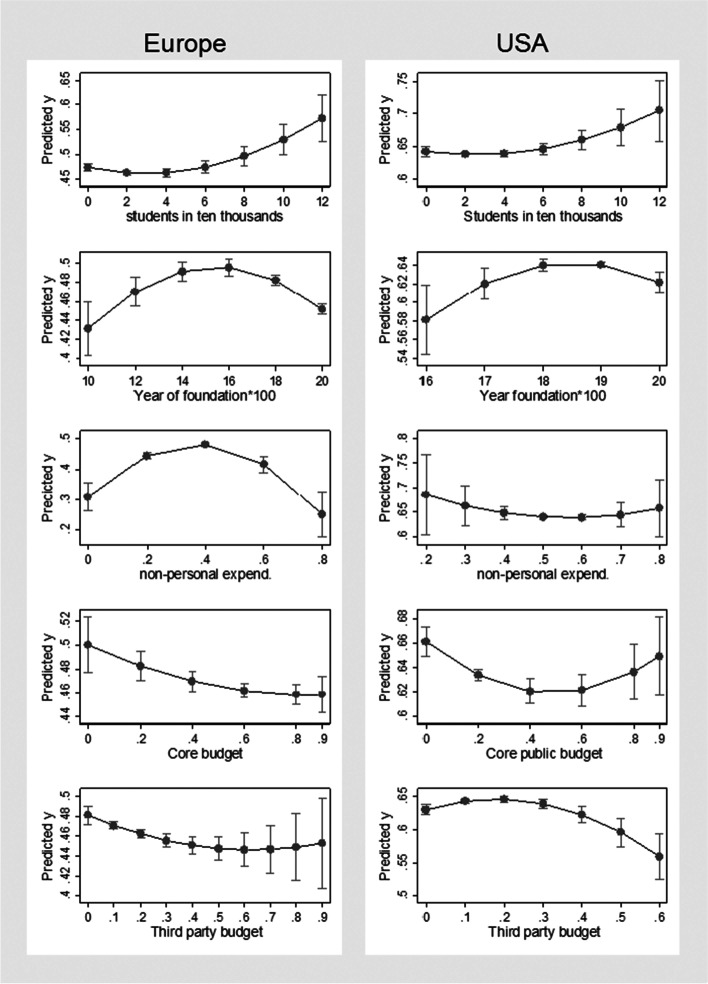
Plots of predicted *Non_acad* at specific values of covariates for Europe and USA. Notes: Predicted y – predicted *Non_acad*, based on the results from Table 3. Source: authors’ own elaboration based on data from ETER and IPEDS

**Fig. 4:**
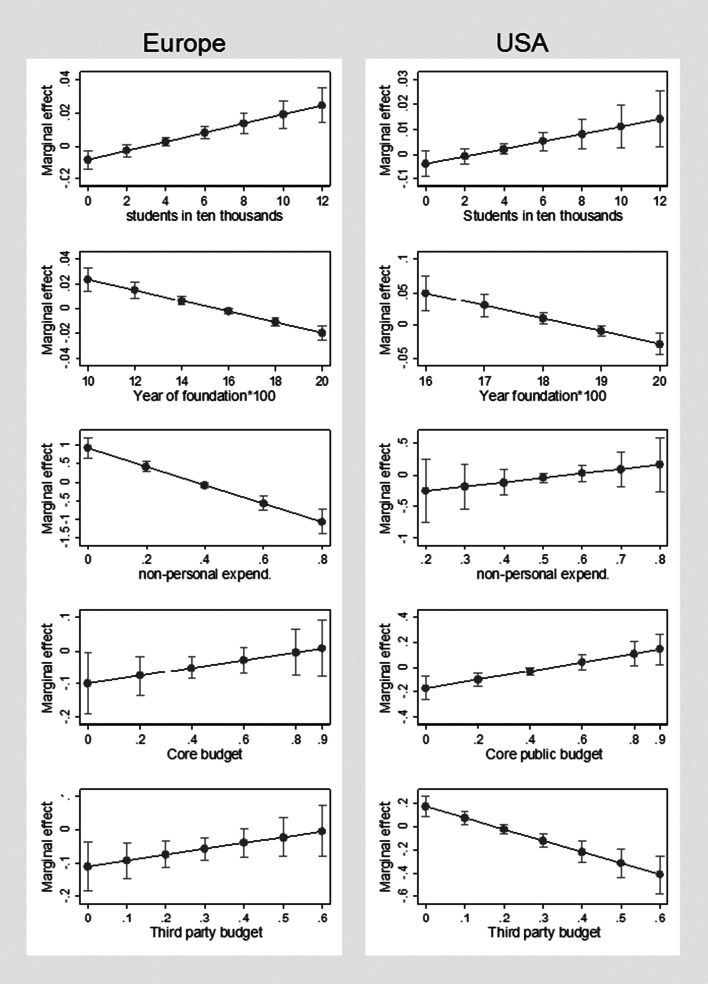
Plots of marginal effects illustrating the results from Table 3 for Europe and USA. Source: authors’ own elaboration based on data from ETER and IPEDS

The results can also be illustrated with plots showing the predicted values of the dependent variable (*Non_acad*) at specified values of covariates and with plots of marginal effects of covariates. For example, the marginal effect of *Non-personnel expenditures (*$Non\_personal)$ on the proportion of non-academic staff (y) is calculated by computing the first derivative of y in Eq. 1 that corresponds to $\frac{\partial Non\_acad}{\partial Non\_personal}={\beta }_{7}+2{\beta }_{8}Non\_personal$. Figure 3 presents the plots of predicted *Non_acad*, while Fig. 4 shows respective marginal effects illustrating the results from Table 3.

The analysis points to a number of similarities between Europe and the USA, but also several noteworthy differences. The results reveal that larger universities—with more students—have a higher proportion of non-academic staff, and for European universities, this trend begins with a negative starting-point relationship, which is quickly reversed. The year of foundation shows an inverted U-shape relationship, which is true for both the European and US samples—the youngest and oldest universities are characterised by a lower share of non-academic staff. Similarly, for the European and American HEIs, private institutions have a lower share of non-academic staff, while HEIs with a higher share of non-academic staff boast a higher number of publications per academic staff. Turning to the potential impact of the funding structure on the share of non-academic staff, there are some interesting results. For the European sample, an inverted U-shape relationship between the share of non-personnel expenditure and *Non_acad* can be observed*.* However, this is not true for the American institutions, for which the relationship is not statistically significant. Furthermore, in the case of the European sample, there is a negative relationship between the share of core funding, while for the American universities, after the initial negative relationship, there is a rebound in the other direction and the nonlinear relationship is confirmed. In contrast, the third-party budget is negatively correlated with the share of non-academic staff in the European HEIs, while in the case of the American sample, the relationship is nonlinear—positive at the beginning and negative for higher shares of third-party budget.

The robustness of our findings was verified in a number of ways. Firstly, to prevent potential endogeneity problems, we run regressions analogous to Eq. (1), in which we considered time-varying independent variables, now including these variables as lagged variables (called lag students, lag Publ_Acad, lag financial variables). The obtained results are presented in Tables S2 (for Europe) and S3 (for the USA) included in the supplementary materials. They generally confirm the aforementioned correlations between the specified variables and the share of non-academic staff. Secondly, we verify the robustness of our findings by adding some extra covariates such as a dummy indicating whether the university has an hospital or whether it is a multisite institution and the number of EU-FP participation (for the European HEIs). In all of these cases, the main findings still hold. In the next step, we rerun the estimation using samples to which different outlier detection methods were applied, and no significant impact on the final results is observed. The obtained results are available as supplementary materials (see Tables S8 and S9). For the European HEIs, we also confirmed the results considering the model specification in relation to the third-party funding variable (with and without its square term) and excluding observations with extremely high values of *Third party*_it_ (Table S10 in the supplementary materials) from the sample. To tackle possible structural multicollinearity that arises in models that include interaction terms, we also ran estimations with mean-centred continuous regressors (Table S11 in the supplementary materials).

## Discussion and further developments

In this work, we attempted to address, with an empirical analysis, a stylised fact observed in many countries of the world: the increase in the number of non-academic staff within higher education institutions. We are aware of the limitations of empirical analyses on this subject linked to the complexity of non-academic staff and the roles that they play in universities. There are, in fact, very few empirical studies on this issue, which is not surprising taking into account the limitation of data available on the subject.

The potential determinants of the share of non-academic staff considered in the quantitative analysis include the number of students enrolled (as a proxy of size of the institution), year of foundation of institution (age of the unit), publication per academic staff member (illustrating university’s research profile) and the share of non-personnel expenditure and budget structure (the share of third-party and core revenues). The choice of the potential determinants was driven by the theoretical background provided in the “Relevant literature” section and by the availability of data described in the “Data and descriptive analysis” section. We perform the comparative analysis of European versus American HEIs, which is a different approach than that in previous studies. The comparison between the two systems reveals that we can expect a higher proportion of non-academic staff in both HEI systems with a sufficiently large number of students. Similarly, the number of publications per academic staff considered a proxy of university’s scientific production and contribution to university rankings and prestige might be a determinant of the proportion of non-academic staff because more active (from a scientific point of view) academic staff might require higher technical and administrative support for handling laboratories and an easier publication process. On the contrary, a lower share of non-academic staff in Europe and the USA can be observed for private HEIs that can capture the impact of organisational and governance differences between public/private units. In the European case, the nonlinear relationship between the share of non-personnel expenditure and the incidence of non-academic staff is confirmed. This result can be a sign that several non-core activities previously performed by non-academic staff are outsourced and conducted outside the institution. At the same time, it should be noted that there is a possibility that previous levels of administrative staff may be a confounding variable and that further research should take this into account.

In both systems (Europe and the USA), it can be observed that the share of non-academic staff reduces when the share of core revenues raises and is sufficiently low, while in the case of the American sample, it increases with the share of core revenues if the latter is sufficiently high. These nonlinear relationships might suggest that there is a certain (superiorly bounded) scale effect, in the sense that low values of the share of core revenues provide HEIs with incentives to increase their organisational efficiency, while for high values of the share of core revenues, these incentives could be jeopardised. Finally, the relationship between the share of third-party revenues and the share of non-academic staff is different for the European and American HEIs. In Europe, the share of non-academic staff goes down as the share of third-party revenues increases. However, in the case of the American sample, it is true only for higher shares of third-party budget.

The increase in third-party funding that decreases the share of non-academic staff in Europe is a problematic result to interpret. This is because we would expect that as third-party funding increases, universities will increase their involvement in third mission activities and would therefore require more technical and administrative support. Over time, this could also lead to an increase in academic staff to support third mission activities involving local councils, industry and other sponsors.

A first rough interpretation of this evidence could be that there is a substantial difference between European and American universities. In Europe, the *Humboldtian* model of university devoted mainly to teaching and research may still be the prevailing one. On the contrary, in the USA, the universities are more innovative and have adopted a more widespread *entrepreneurial* model, through which they are better able to be active in the sphere of third mission activities. This could explain the observed differences, although a more in-depth analysis would be required to more accurately interpret the results obtained and this is left to future work.

Third-party revenues may include different lines of funds, such as due to projects commissioned by public agencies or private companies as well as due to large projects involving several universities or to small ones. Therefore, the overall impact of the third-party revenues on the non-academic staff may be complex to analyse and would require much more unbundled data to be study in-depth. For instance, when new researchers are hired and they activate new research projects, the impact on non-academic staff may be very different. On one hand, in the case projects are not too big and their duration is short, non-academic personnel of the department may be asked to work overtime (instead of assuming new people), or temporary office workers are paid to provide technical and administrative assistance for the projects, or outsourced professional services are purchased to manage projects. In all these cases, the share of third-party budget raises while the share of non-academic staff reduces (as the measured non-academic staff essentially remains constant). On the other hand, in the case whereby projects are big and their duration is years long, additional non-academic units could be hired. The different mix of research projects and different strategies in terms of insourcing or outsourcing activities to manage projects could also partially explain the difference among European and American HEIs.

Our analysis focuses on the evaluation of the potential determinants of the share of non-academic staff in HEIs, but the presented results serve as a clear indication that some determinants of the incidence of non-academic staff, which include size, prestige, year of foundation and financial structure of the universities, are usually considered to be performance measures for HEIs. This evidence may provide a motivation for investigating the relationship between non-academic staff and university performance. However, there are conceptual, methodological and critical data problems to be addressed with regard to this further relevant issue (mainly oversimplifying academic versus non-academic personnel data representation in HEIs).

The impact of non-academic staff on university performance may be difficult to evaluate due to the participation of the faculty in the complex decision-making process in universities. For instance, McCormick and Meiners (1988) find that involving administrative personnel in the decision-making process is usually associated with better university performance, while faculty participation may lead to low-quality decisions. Kaplan (2004) observes that faculty participation in academic issues yields decisions which are not beneficial to the university, being made in the interest of faculty members instead. Brown (2001) carries out more in-depth analyses and finds that faculty participation in the academic decision-making process may lead to high-quality decisions if financial decisions and day-to-day management are under the exclusive control of administrative personnel. In particular, Cunningham (2009) reveals that faculty participation in academic decisions is correlated with better performance only when it concerns faculty personnel and student matters. Brown (2014) analyses a potential relationship between university board characteristics (an important segment of non-academic staff) and university performance through quantitative data related to the US universities. On one side, Brown’s findings confirm that the size and composition of the board are affected by university size, religious affiliation and university type (liberal arts colleges versus institutions that offer doctoral degrees). On the other side, the results show that a larger size of the board and a higher fraction of the board chosen directly by alumni have a positive impact on university performance.

The results obtained in this paper point to the presence of several nonlinear relationships and for this reason, we think that further research should employ nonparametric methods (Daraio, 2018) to accomplish the difficult task of modelling the impact of non-academic staff on performance. In addition, investing in European data appears timely. The collection and integration of more detailed information on the composition of non-academic staff in European HEIs—taking into account the information available for the USA—could be another step towards a better understanding of both the determinants of the share of non-academic staff and the complex relationship existing between non-academic staff and university performance. With the results obtained in this paper, we can surmise that the determinants of the share of non-academic staff vary depending on the role that different non-academic staff categories play in the performance of HEIs.

An interesting extension of this piece of research could be the estimation of dynamic models including the lagged dependent variable given that previous levels of non-academic staff may drive subsequent levels. The investigation of this relationship would be of key interest for HE researchers in the field. At present, longitudinal data available do not provide us with a sufficient number of years to carry out such analysis, so future research will be required when additional data will be available.

Finally, in future studies, it will be interesting to analyse the impact of COVID-19 on the functioning of HEIs. The COVID-19 pandemic combined with introduced restrictions and lockdowns affected the functioning of HEIs in many aspects such as closing units, moving teaching and learning online and slowing down internationalisation and introduction of working from home for their staff (Marinoni et al., 2020). Additionally, in view of the possible budget cuts (Blankenberger & Williams, 2020) not only in relation to public funds but also private revenues (e.g. drop in tuition fees), HEIs, in the long run, will have to adjust to a new financial constraint also with a possible restructuration of the employment structure (e.g. Burki, 2020 reports for HEIs in the UK that the most unfavourable situation is for short-term contracted staff and PhDs whose funding is precarious). Needless to say, it would be very interesting to verify how COVID-19 impacts not only the model of work (teaching and learning), but also other aspects of HEI functioning such as the structure of employment in HEIs and possible pressure on employment cuts, on which there is not much evidence yet.^26^

## Concluding remarks

Our investigation focuses on a neglected topic, i.e. the determining factors concerning non-academic staff of HEIs. Considering the new public management accountancy needs and the scarce resources of universities everywhere, this is a very important subject from a policy-making point of view, on which many more quantitative and systematic analyses are needed. Building and extending the existing scant literature on the topic, we examine two unique datasets to provide updated empirical evidence and an indirect *comparative* analysis on the structure and heterogeneity of HEIs in Europe and the USA. Our analyses shed some light on the determinants of the share (incidence) of non-academic staff, which include size, prestige, year of foundation and the financial structure of the analysed HEIs. Interestingly, these determinants are typically considered proxies of HEI performance. The empirical results reported in this paper can therefore be considered the first step towards a more extended analysis to try to understand the relationship existing between non-academic staff and university performance. To address this relevant research question, conceptual, methodological and data problems should be analysed further and deeper. In this paper, we provide some updated empirical evidence that offers certain insights to facilitate thorough investigations and further research into the matter at hand.

An interesting extension of this piece of research could be the estimation of dynamic models including the lagged dependent variable given that previous levels of non-academic staff may drive subsequent levels. The investigation of this relationship would be of key interest for HE researchers in the field. At present, longitudinal data available do not provide us with a sufficient number of years to carry out such analysis, so future research will be required when additional data will be available.

### Electronic supplementary material

Below is the link to the electronic supplementary material.

Supplementary file1 (DOCX 69 KB)

## Data Availability

Data on European and US HEIs come from openly available sources: European Tertiary Education Register project (https://eter-project.com/) and Integrated Postsecondary Education Dataset (https://nces.ed.gov/ipeds/). The bibliometric data can be obtained for research purposes from the Centre for Science and Technology Studies (CWTS) at University of Leiden through the EU-FP RISIS2 project (https://rcf.risis2.eu/datasets).

## Appendix

**Table 4:** The final European sample—the number of universities by country

Country	2011	2012	2013	2014	2015	2016	Total
AT	19	19	19	19	19	19	114
BE	6	6	6	6	6	6	36
BG	11	11	11	11	11	11	66
CH	12	12	12	12	12	12	72
CY	1	1	1	1	1	1	6
CZ	22	22	22	22	22	22	132
DE	84	84	84	84	84	84	504
DK	8	8	8	8	8	8	48
EE	4	4	4	4	4	4	24
ES	69	69	69	69	69	69	414
FR	62	62	62	62	62	62	372
GR	18	18	18	18	18	18	108
HR	5	5	5	5	5	5	30
IE	7	7	7	7	7	7	42
IS	2	2	2	2	2	2	12
IT	69	69	69	69	69	69	414
LI	1	1	1	1	1	1	6
LT	12	12	12	12	12	12	72
MT	1	1	1	1	1	1	6
NL	13	13	13	13	13	13	78
NO	8	8	8	8	8	8	48
PL	60	60	60	60	60	60	360
PT	17	17	17	17	17	17	102
SE	27	27	27	27	27	27	162
SK	18	18	18	18	18	18	108
UK	119	119	119	119	119	119	714
Total	675	675	675	675	675	675	4050

Source: own elaboration based on ETER data.

**Table 5:** The share of non-academic staff to total staff across countries over years, country-average

Country	2011	2012	2013	2014	2015	2016
AT	0.37	0.39	0.38	0.38	0.36	0.37
BE	0.30	0.30	0.29	0.29	0.30	0.30
BG	0.38	0.38	0.38	0.41	0.41	0.41
CH	0.32	0.32	0.32	0.32	0.32	0.32
CY	0.59	0.57	0.57	0.58	0.58	0.56
CZ	0.42	0.43	0.44	0.44	0.46	0.46
DE	0.47	0.47	0.47	0.47	0.47	0.47
DK	0.43	0.42	0.40	0.38	0.37	0.36
EE	0.42	0.42	0.42	0.43	0.46	0.47
ES	0.41	0.41	0.41	0.41	0.39	0.40
FR	0.54	0.53	0.52	0.53	0.52	0.53
GR	0.24	0.22	0.20	0.20	0.17	0.16
HR	0.39	0.41	0.42	0.43	0.43	0.44
IE	0.46	0.45	0.47	0.45	0.46	0.45
IS	0.76	0.76	0.76	0.77	0.79	0.80
IT	0.37	0.37	0.38	0.39	0.39	0.38
LI	0.47	0.51	0.54	0.44	0.45	0.46
LT	0.51	0.53	0.53	0.53	0.52	0.52
MT	0.49	0.47	0.46	0.44	0.44	0.42
NL	0.42	0.42	0.42	0.41	0.41	0.41
NO	0.41	0.42	0.42	0.42	0.42	0.41
PL	0.45	0.45	0.45	0.45	0.45	0.44
PT	0.46	0.46	0.46	0.45	0.46	0.45
SE	0.42	0.42	0.42	0.42	0.41	0.41
SK	0.46	0.47	0.46	0.46	0.46	0.46
UK	0.55	0.54	0.54	0.53	0.53	0.53
Mean	0.45	0.45	0.45	0.45	0.45	0.45

Source: own elaboration based on data from ETER.

**Table 6:** Partial correlation of covariates used in the analysis—European sample

	Students_it_	YearFound_i_	Private_i_	Publ_Acad_it_	Non_personal_it_	Core budget_it_
Students_it_	1.000					
YearFound_i_	− 0.437	1.000				
Private_i_	− 0.269	0.166	1.000			
Publ_Acad_it_	0.247	− 0.211	− 0.157	1.000		
Non_personal_it_	− 0.051	0.007	0.250	− 0.082	1.000	
Core_budget_it_	0.096	− 0.032	− 0.359	− 0.031	− 0.259	1.000
Third_party_it_	− 0.022	− 0.132	− 0.022	0.348	0.014	− 0.235

Source: own elaboration based on data from ETER.

**Table 7:** Partial correlation of variables used in the analysis—US sample

	Students_it_	YearFound_i_	Private_i_	Publ_Acad_it_	Non_personal_it_	Core budget_it_
Students_it_	1.000					
YearFound_i_	− 0.066	1.000				
Private_i_	− 0.440	− 0.156	1.000			
Publ_Acad_it_	0.281	− 0.364	− 0.051	1.000		
Non_personal_it_	0.099	0.027	− 0.243	0.150	1.000	
Core_budget_it_	0.194	− 0.010	− 0.521	0.253	0.310	1.000
Third_party_it_	0.266	− 0.195	− 0.314	0.577	0.043	0.115

Source: own elaboration based on data from IPEDS.

**Table 8:** Determinants of *Non_acad* (the dependent variable: the ratio of non-academic staff to total staff), European sample

	(1)	(2)	(3)	(4)	(5)	(6)	(7)
Students_it_	− 0.008***	− 0.007**	− 0.009***	− 0.007***	− 0.007***	− 0.007***	− 0.007**
	[0.002]	[0.003]	[0.003]	[0.003]	[0.003]	[0.003]	[0.003]
Students_it_^2^	0.001***	0.001***	0.002***	0.001***	0.001***	0.001***	0.001***
	[0.000]	[0.000]	[0.000]	[0.000]	[0.000]	[0.000]	[0.000]
YearFound_i_	0.076***	0.077***	0.072***	0.079***	0.082***	0.078***	0.078***
	[0.011]	[0.012]	[0.012]	[0.012]	[0.012]	[0.012]	[0.012]
YearFound_i_^2^	− 0.002***	− 0.002***	− 0.002***	− 0.003***	− 0.003***	− 0.002***	− 0.003***
	[0.000]	[0.000]	[0.000]	[0.000]	[0.000]	[0.000]	[0.000]
Private_i_	− 0.008	− 0.015	− 0.007	− 0.024*	− 0.031**	− 0.015	− 0.017
	[0.008]	[0.013]	[0.013]	[0.014]	[0.014]	[0.013]	[0.013]
Publ_Acad_it_	0.013***	0.021***	0.019***	0.022***	0.023***	0.025***	0.026***
	[0.004]	[0.004]	[0.004]	[0.004]	[0.004]	[0.004]	[0.004]
Non_personal_it_		0.002	0.795***				
		[0.035]	[0.126]				
Non_personal_it_^2^			− 1.033***				
			[0.169]				
Core_budget_it_				− 0.016	− 0.098**		
				[0.014]	[0.043]		
Core_budget_it_^2^					0.081**		
					[0.039]		
Third_party_it_						− 0.036**	− 0.074**
						[0.018]	[0.036]
Third_party_it_^2^							0.061
							[0.060]
N	4050	2742	2742	2902	2902	2710	2710
No countries	26	21	21	21	21	20	20
R2	0.47	0.43	0.44	0.43	0.43	0.44	0.44

**p* < 0.10, ***p* < 0.05, ****p* < 0.01, country and time fixed effects included (not reported). Robust standard errors. Specifications (2)–(5), no data on BG, ES, GR, HR, IS; specifications (6)–(7), additionally, no data on CZ.

Source: own elaboration based on data from ETER.

**Table 9:** Determinants of *Non_acad* (the dependent variable: the ratio of non-academic staff to total staff), US sample

	(1)	(2)	(3)	(4)	(5)	(6)	(7)
Students_it_	− 0.002	− 0.002	− 0.002	− 0.003	− 0.001	− 0.002	− 0.004
	[0.003]	[0.003]	[0.003]	[0.003]	[0.003]	[0.003]	[0.003]
Students_it_^2^	0.001*	0.001*	0.001*	0.001**	0.001	0.001*	0.001**
	[0.000]	[0.000]	[0.000]	[0.000]	[0.000]	[0.000]	[0.000]
YearFound_i_	0.330***	0.310***	0.310***	0.313***	0.295***	0.351***	0.373***
	[0.100]	[0.100]	[0.100]	[0.097]	[0.098]	[0.102]	[0.097]
YearFound_i_^2^	− 0.009***	− 0.008***	− 0.008***	− 0.008***	− 0.008***	− 0.010***	− 0.010***
	[0.003]	[0.003]	[0.003]	[0.003]	[0.003]	[0.003]	[0.003]
Private_i_	− 0.002	− 0.004	− 0.004	− 0.014**	− 0.027***	− 0.004	− 0.001
	[0.004]	[0.004]	[0.004]	[0.007]	[0.010]	[0.004]	[0.004]
Publ_Acad_it_	0.053***	0.054***	0.054***	0.052***	0.051***	0.055***	0.053***
	[0.003]	[0.003]	[0.003]	[0.003]	[0.003]	[0.004]	[0.004]
Non_personal_it_		− 0.043	− 0.047				
		[0.032]	[0.333]				
Non_personal_it_^2^			0.005				
			[0.309]				
Core_ budget_it_				− 0.029*	− 0.119**		
				[0.015]	[0.048]		
Core_budget_it_^2^					0.118**		
					[0.058]		
Third_party_it_						− 0.018	0.166***
						[0.020]	[0.044]
Third_party_it_^2^							− 0.425***
							[0.099]
N	2046	2042	2042	2042	2042	2042	2042
R2	0.41	0.41	0.41	0.41	0.41	0.41	0.42

**p* < 0.10, ***p* < 0.05, ****p* < 0.01, state and time fixed effects included (not reported). Robust standard errors.

Source: own elaboration based on data from IPEDS.

## Footnotes

1 In our empirical analysis, we will refer to the simple binary division (academic versus non-academic staff), which is still the predominant division in many universities and continues to be an important categorisation, e.g. as far as distinct contractual terms for academic/non-academic staff are considered. Additionally, the binary division makes the operationalisation of different aspects of HEIs easier. However, it should be underlined that the division of HEI personnel into specific categories is a complex issue and the binary division is even more problematic especially if one takes into account: (a) development of administrative roles in universities, with administrative work becoming more and more professional, (b) conducting part of administrative work by academic staff with a double role, e.g. deans/heads of departments/faculties who keep their academic position and manage units at the same time. See the “Relevant literature” section for a wider discussion about the nomenclature and heterogeneity of non-academic staff.

2 Specifically, he writes: “For many of these career managers, promoting teaching and research is less important than expanding their own administrative domains. Under their supervision, the means have become the end.” (Grinsberg, 2011 p. 2).

3 See Pruvot and Estermann (2018). In the UK, for instance, race, disability and gender equality duties may have an impact on universities’ professional staff (Baltaru 2019b).

4 Furthermore, specific missing data in ETER were imputed (see the “Data and descriptive analysis” section).

5 This group of non-academic professionals mainly consists of senior administrators such as, for instance, the Director of Operations Management, Director of Quality Assurance, Director of Educational and Student Affairs or Head of Strategic Projects. Together with several academics holding managerial positions, these high professional administrators represent a third organisational domain that has developed to ensure better coordination and organisation of the activities of other academic and non-academic employees in order to balance their different—and sometimes conflicting—interests with the pursue of valuable outcomes for HEIs (Kallenberg 2018; Smith et al., 2021; Whitchurch 2007).

6 Baltaru and Soysal (2018) investigate several different factors, but they do not find empirical evidence that the proportion of non-academic staff to total staff depends on student enrolments, budget cuts or deregulation.

7 According to some authors, this conflicting relationship does not necessarily jeopardise university outcomes (Bacon, 2009).

8 Additionally, some data concerning French HEIs are provided for 2017.

9 The bibliometric data can be obtained for research purposes through the EU-FP RISIS2 project (https://rcf.risis2.eu/datasets). We would like to thank Josephine Bergmans and Ed Noyons from the CWTS, Leiden University, for providing the data.

10 The restriction is based on the variable, *Institution category standardised* in ETER, which classified institutions into three groups: universities, university of applied sciences/colleges and others.

11 The *Distance education institution* variable in ETER includes three categories: no or limited amount of distance education (< 20% of students of distance programmes), a substantial share of distance education and mostly distance education (> 90% of students of distance programmes). The mean value of the number of students for the distance education institutions reported in ETER is 30,000 and the mean number of students per FTE academic staff is 78.5. When comparing these values with the statistics reported in Table 2, clear differences between distance and non-distance institutions can be observed. These differences demand a separate analysis of these two types of institutions.

12 The imputation method is based on Bruni et al. (2021) from which the imputed data were taken.

13 Previous studies included in the relevant literature consider the number of non-academics relative to the number of academics/total staff (Baltaru and Soysal 2018; Gornitzka and Larsen 2004).

14 The initial data examination showed that there is an enormous variation in the proportion of non-academic staff of the analysed institutions. We identified institutions with a proportion of non-academic staff equal to zero as well as institutions with an extremely high proportion. For example, there are three universities reporting zero non-academic staff, namely Webster University Vienna, a private university in Austria; Link Campus University in Italy; and International Hellenic University in Greece. On the other hand, the universities with the highest proportion of non-academic staff, i.e. above 85%, include London Business School in the UK, Campus Bio-Medico University in Italy and International University of Andalucía in Spain. For this reason, to ensure that the results were not driven by outliers, we decided to eliminate these extreme observations by considering those institutions with *Non_acad* either lower than 0.1 or higher than 0.85 as outliers. However, to check the robustness of these identified thresholds, we repeated the empirical analysis considering two alternative methods of outlier detection as well. The first method is based on percentiles (and identifies outliers as those observations that are either smaller than the 1st percentile or larger than the 99th percentile), while the other considers the distance from the mean (considering those observations that have values of *Non_acad* three standard deviations away from the mean as outliers). As described in the “Results” section, the choice of the methodology for detecting outliers does not affect our empirical results.

15 Using IPEDS, FTE for each of the occupational category is derived by adding the full-time staff headcount to 1/3 of the part-time headcount for each occupational category.

16 ETER provides the classification of staff in binary format: academic versus non-academic staff (giving total staff when summed up). Non-academic staff includes HEI-level management staff such as the director, administrative director and head of service; HEI-level administrative staff, including both central-level and department-level staff; staff engaged in maintenance and operations, including special services such as IT (Information Technologies); and undergraduate students employed for teaching assistance or research.

17 In the case of Europe, core revenues refer to funding available for the operations of the institution as a whole, which are not earmarked for specific activities and whose internal allocation can be decided fairly freely by the institution itself. They are further divided into basic government allocation and other revenues, source: ETER (2018). For the USA, they are comprised of federal, state and local appropriations and federal, state and non-operating grants; for public institutions: according to accounting standards established by the Governmental Accounting Standards Board (GASB), for private not-for-profit: according to the Financial Accounting Standards Board (FASB), source: IPEDS (2020).

18 In the case of Europe, third-party revenues refer to funds for specific activities and institutional units; they are the sum of grants from national and international funding agencies for research activities; funds from charities and non-profit organisations for specific research and educational purposes; contracts concluded with public bodies, non-profit organisations and private companies for specific research and services; fees/payments from companies for educational services and research; and service grants from companies, source: ETER (2018). For the USA, in the case of public institutions: federal, state, and local operating grants and contracts, in the case of private not-for-profit: federal, state, and local grants and contracts, source: IPEDS (2020).

19 In Europe, non-personnel expenditure includes expenditure on goods and services other than staff compensation, source: ETER (2018). In USA, all expenses not included in salaries and wages as compensation for services to all employees, source: IPEDS (2020).

20 See Table S1 in the supplementary materials for details about the mapping of core and third-party revenues of European and US HEIs derived from Lepori et al. (2019) and applied in this paper.

21 This variable also includes expenditures different from outsourced activities.

22 It would be very difficult to merge the two datasets and treat them as a unique dataset due to definitional and comparability problems between the variables.

23 In the case of European HEIs, there are some outliers for third-party funding with extremely high values, e.g. third-party funding being higher than 90% (see also statistics in Table 2). To check the robustness of our results and to be sure that the results of our empirical analysis are not driven by those outliers, we rerun estimations of the analyses without those observations. We find that the results still hold. See Table S10 in the supplementary materials.

24 Following the suggestion of a reviewer, we attempted to estimate a dynamic model with a lagged dependent variable using an appropriate two-step generalised method of moments (GMM). For an introduction and more details on GMM, see e.g. Mátyás et al. (1999). However, it turned out that the introduced lagged dependent variable, which was highly significant, absorbed all the effects of the independent variables in which we are interested. It is most likely that the introduced lagged value of the share of non-academic staff to total staff captured the effects of past independent variables mainly due to a very persistent dependent variable. This is a well-known problem in the literature when autoregressive parameters dominate regression and swap other independent variables (see e.g. Keele and Kelly 2006). This problem is also aggravated by (i) a very persistent dependent variable (see Table 5 in the Appendix), (ii) the short time period of our analysis and (iii) a rather demanding structure of the polynomial model. Additional details and the obtained results are available from the authors upon request. Even though we do not possess data covering a sufficient number of years to carry out dynamic analysis yet, it would be an interesting extension of the study when additional data will be collected and available.

25 To tackle possible structural multicollinearity that arises in models that include interaction terms, we also run estimations with mean-centred continuous regressors. The results are presented in Table S11 of the supplementary materials.

26 Changes at the level of individual HEIs will also depend on how the governments of different countries have responded to the COVID-19 crisis in terms of university funding.

## Code availability

Upon request.
