# Supplementary material for: Determinants of the incidence of non-academic staff in European and US HEIs
Source: High Educ (Dordr). 2022 Feb 17;85(1):55–83. doi: 10.1007/s10734-022-00819-7 (PMC8853347; doi:10.1007/s10734-022-00819-7)
Supplement: Supplementary file 1 — Supplementary file1 (DOCX 69 KB) [file 10734_2022_819_MOESM1_ESM.docx]

**Supplementary materials not to be included in the main text of the manuscript**

**Table S1. Mapping scheme for HEI revenues**

|  | **US HEIs** | | **European HEIs** |
| --- | --- | --- | --- |
| **Variable** | **IPEDS public HEIs** | **IPEDS private non profit HEIs** | **ETER** |
| Core Budget (public) | Federal Appropriations | | Basic government allocation (central or regional) |
|  | State Appropriations | |  |
|  | Local Appropriation, Education District taxes, and Similar Support | Local Appropriations |  |
|  | Federal Non Operating Grants |  |  |
|  | State Non Operating Grants |  |  |
|  | Local Non Operating Grants |  |  |
| Core budget (private) | Gift (Including Contributions from affiliate Organizations) | Private gifts | Gifts and donations |
|  | Other Revenues and Additions |  |  |
|  | Other Non Operating Revenues |  |  |
|  | Other sources - operating |  | Interests |
|  | Investment Income | Investment Return | Investment income |
| Third Party | Federal Operating Grants and Contracts | Federal grants and contracts | Public grants and contracts (central, regional ,local) |
|  | State Operating Grants and Contracts | State grants and contracts |  |
|  | Local Operating Grants and Contracts | Local grants and contracts | Grants and contracts from abroad |
|  | Private Operating Grants and Contracts | Private gifts, grants, and contracts | Private grants and contracts |

Source: Lepori et al. (2019) Supporting information Table S1

**Table S2. Determinants of *Non_acad* (the dependent variable: the ratio of non-academic staff to total staff), the European sample, time-varying independent variables included as lags**

|  | (1) | (2) | (3) | (4) | (5) | (6) | (7) | (8) |
| --- | --- | --- | --- | --- | --- | --- | --- | --- |
| Students_it-1_ | -0.007*** | -0.006** | -0.008*** | -0.007** | -0.006** | -0.007** | -0.006** | -0.007** |
|  | [0.003] | [0.003] | [0.003] | [0.003] | [0.003] | [0.003] | [0.003] | [0.003] |
| Students_it-1_^2^ | 0.001*** | 0.001*** | 0.001*** | 0.001*** | 0.001*** | 0.001*** | 0.001*** | 0.001*** |
|  | [0.000] | [0.000] | [0.000] | [0.000] | [0.000] | [0.000] | [0.000] | [0.000] |
| YearFound_i_ | 0.077*** | 0.077*** | 0.073*** | 0.078*** | 0.083*** | 0.078*** | 0.079*** | 0.070*** |
|  | [0.012] | [0.013] | [0.012] | [0.013] | [0.013] | [0.012] | [0.013] | [0.013] |
| YearFound_i_ ^2^ | -0.002*** | -0.002*** | -0.002*** | -0.002*** | -0.003*** | -0.003*** | -0.003*** | -0.002*** |
|  | [0.000] | [0.000] | [0.000] | [0.000] | [0.000] | [0.000] | [0.000] | [0.000] |
| Private_i_ | -0.009 | -0.024* | -0.016 | -0.036** | -0.045*** | -0.022* | -0.023* | -0.043*** |
|  | [0.008] | [0.014] | [0.014] | [0.015] | [0.015] | [0.014] | [0.014] | [0.016] |
| Publ_Acad_it-1_ | 0.009** | 0.018*** | 0.016*** | 0.019*** | 0.020*** | 0.022*** | 0.023*** | 0.021*** |
|  | [0.004] | [0.004] | [0.004] | [0.004] | [0.004] | [0.004] | [0.004] | [0.005] |
| Non_personal_it-1_ |  | 0.009 | 0.723*** |  |  |  |  | 0.868*** |
|  |  | [0.038] | [0.146] |  |  |  |  | [0.158] |
| Non_personal_it-1_^2^ |  |  | -0.933*** |  |  |  |  | -1.171*** |
|  |  |  | [0.201] |  |  |  |  | [0.219] |
| Core budget_it-1_ |  |  |  | -0.026* | -0.133*** |  |  | -0.144*** |
|  |  |  |  | [0.015] | [0.049] |  |  | [0.054] |
| Core budget_it-1_^2^ |  |  |  |  | 0.106** |  |  | 0.095* |
|  |  |  |  |  | [0.044] |  |  | [0.053] |
| Third party_it-1_ |  |  |  |  |  | -0.024 | -0.056 | -0.084** |
|  |  |  |  |  |  | [0.018] | [0.038] | [0.040] |
| Third party_it-1_^2^ |  |  |  |  |  |  | 0.054 | 0.061 |
|  |  |  |  |  |  |  | [0.062] | [0.060] |
| N | 3375 | 2387 | 2387 | 2507 | 2507 | 2383 | 2383 | 2244 |
| No countries | 26 | 21 | 21 | 21 | 21 | 20 | 20 | 20 |
| R^2^ | 0.47 | 0.43 | 0.44 | 0.43 | 0.43 | 0.43 | 0.43 | 0.46 |

Notes: * p<0.10, ** p<0.05, *** p<0.01, Country and time fixed effects included (not reported). Robust standard errors. Specifications (2)- (5): no data on BG, ES, GR, HR, IS, specification (6)-(8): additionally, no data on CZ

Source: Source: own elaboration based on data from ETER

**Table S3. Determinants of *Non_acad* (the dependent variable: the ratio of non-academic staff to total staff), the US sample, time-varying independent variables included as lags**

|  | (1) | (2) | (3) | (4) | (5) | (6) | (7) | (8) |
| --- | --- | --- | --- | --- | --- | --- | --- | --- |
| Students_it-1_ | -0.002 | -0.002 | -0.003 | -0.003 | -0.002 | -0.002 | -0.004 | -0.004 |
|  | [0.003] | [0.003] | [0.003] | [0.003] | [0.003] | [0.003] | [0.003] | [0.003] |
| Students_it-1_^2^ | 0.001** | 0.001** | 0.001** | 0.001** | 0.001* | 0.001** | 0.001*** | 0.001** |
|  | [0.000] | [0.000] | [0.000] | [0.000] | [0.000] | [0.000] | [0.000] | [0.000] |
| YearFound_i_ | 0.321** | 0.299** | 0.298** | 0.300** | 0.281** | 0.331*** | 0.356*** | 0.337*** |
|  | [0.129] | [0.129] | [0.129] | [0.125] | [0.125] | [0.125] | [0.120] | [0.118] |
| YearFound_i_ ^2^ | -0.009** | -0.008** | -0.008** | -0.008** | -0.008** | -0.009*** | -0.010*** | -0.009*** |
|  | [0.003] | [0.003] | [0.003] | [0.003] | [0.003] | [0.003] | [0.003] | [0.003] |
| Private_i_ | -0.003 | -0.005 | -0.005 | -0.016** | -0.029*** | -0.004 | -0.001 | -0.037*** |
|  | [0.004] | [0.004] | [0.004] | [0.008] | [0.011] | [0.004] | [0.004] | [0.011] |
| Publ_Acad_it-1_ | 0.051*** | 0.051*** | 0.051*** | 0.049*** | 0.048*** | 0.052*** | 0.050*** | 0.049*** |
|  | [0.004] | [0.004] | [0.004] | [0.004] | [0.004] | [0.005] | [0.005] | [0.005] |
| Non_personal_it-1_ |  | -0.036 | 0.3 |  |  |  |  | -0.093 |
|  |  | [0.037] | [0.409] |  |  |  |  | [0.543] |
| Non_personal_it-1_^2^ |  |  | -0.316 |  |  |  |  | 0.072 |
|  |  |  | [0.382] |  |  |  |  | [0.517] |
| Core budget_it-1_ |  |  |  | -0.032* | -0.125** |  |  | -0.179*** |
|  |  |  |  | [0.017] | [0.055] |  |  | [0.055] |
| Core budget_it-1_^2^ |  |  |  |  | 0.123* |  |  | 0.183*** |
|  |  |  |  |  | [0.067] |  |  | [0.067] |
| Third party_it-1_ |  |  |  |  |  | -0.012 | 0.186*** | 0.195*** |
|  |  |  |  |  |  | [0.022] | [0.051] | [0.052] |
| Third party_it-1_^2^ |  |  |  |  |  |  | -0.454*** | -0.509*** |
|  |  |  |  |  |  |  | [0.111] | [0.115] |
| N | 1705 | 1701 | 1701 | 1701 | 1701 | 1701 | 1701 | 1701 |
| R^2^ | 0.4 | 0.4 | 0.4 | 0.4 | 0.4 | 0.4 | 0.41 | 0.42 |

Notes: * p<0.10, ** p<0.05, *** p<0.01, State and time fixed effects included (not reported). Robust standard errors.

Source: own elaboration based on data from IPEDS

**Table S4. Determinants of *Non_acad* (additional RHS variable: multisite institution), European sample**

|  | (1) | (2) | (3) | (4) | (5) | (6) | (7) | (8) |
| --- | --- | --- | --- | --- | --- | --- | --- | --- |
| Multisite_i_ | -0.001 | 0.004 | 0.003 | 0.005 | 0.005 | 0.006* | 0.006* | 0.004 |
|  | [0.003] | [0.003] | [0.003] | [0.003] | [0.003] | [0.004] | [0.004] | [0.004] |
| Students_it_ | -0.007*** | -0.007** | -0.010*** | -0.008*** | -0.008*** | -0.008*** | -0.007*** | -0.009*** |
|  | [0.002] | [0.003] | [0.003] | [0.003] | [0.003] | [0.003] | [0.003] | [0.003] |
| Students_it_^2^ | 0.001*** | 0.001*** | 0.002*** | 0.001*** | 0.001*** | 0.001*** | 0.001*** | 0.001*** |
|  | [0.000] | [0.000] | [0.000] | [0.000] | [0.000] | [0.000] | [0.000] | [0.000] |
| YearFound_i_ | 0.076*** | 0.077*** | 0.073*** | 0.080*** | 0.083*** | 0.079*** | 0.080*** | 0.068*** |
|  | [0.011] | [0.012] | [0.012] | [0.012] | [0.012] | [0.012] | [0.012] | [0.012] |
| YearFound_i_ ^2^ | -0.002*** | -0.002*** | -0.002*** | -0.003*** | -0.003*** | -0.003*** | -0.003*** | -0.002*** |
|  | [0.000] | [0.000] | [0.000] | [0.000] | [0.000] | [0.000] | [0.000] | [0.000] |
| Private_i_ | -0.008 | -0.017 | -0.008 | -0.026* | -0.034** | -0.017 | -0.019 | -0.030** |
|  | [0.008] | [0.013] | [0.013] | [0.014] | [0.014] | [0.013] | [0.013] | [0.014] |
| Publ_Acad_it_ | 0.013*** | 0.021*** | 0.019*** | 0.022*** | 0.023*** | 0.025*** | 0.026*** | 0.024*** |
|  | [0.004] | [0.004] | [0.004] | [0.004] | [0.004] | [0.004] | [0.004] | [0.004] |
| Non_personal_it_ |  | 0.003 | 0.794*** |  |  |  |  | 0.917*** |
|  |  | [0.034] | [0.125] |  |  |  |  | [0.136] |
| Non_personal_it_^2^ |  |  | -1.031*** |  |  |  |  | -1.237*** |
|  |  |  | [0.169] |  |  |  |  | [0.184] |
| Core budget_it_ |  |  |  | -0.017 | -0.100** |  |  | -0.101** |
|  |  |  |  | [0.014] | [0.043] |  |  | [0.047] |
| Core budget_it_^2^ |  |  |  |  | 0.083** |  |  | 0.061 |
|  |  |  |  |  | [0.039] |  |  | [0.047] |
| Third party_it_ |  |  |  |  |  | -0.036** | -0.075** | -0.111*** |
|  |  |  |  |  |  | [0.018] | [0.036] | [0.037] |
| Third party_it_^2^ |  |  |  |  |  |  | 0.062 | 0.089 |
|  |  |  |  |  |  |  | [0.060] | [0.057] |
| N | 4039 | 2742 | 2742 | 2902 | 2902 | 2710 | 2710 | 2570 |
| R^2^ | 0.47 | 0.43 | 0.44 | 0.43 | 0.43 | 0.44 | 0.44 | 0.46 |

Notes: * p<0.10, ** p<0.05, *** p<0.01, Country and time fixed effects included (not reported). Robust standard errors. Specifications (2)- (5): no data on BG, ES, GR, HR, IS, specification (6)-(8): additionally, no data on CZ. Multisite: 1-multisite institution, 0 –single site; multisite institution defined as HEI having other establishments at the local, sub-regional levels (NUTS 3).

Source: own elaboration based on data from ETER

**Table S5. Determinants of *Non_acad* (additional RHS variable: university hospital), European sample**

|  | (1) | (2) | (3) | (4) | (5) | (6) | (7) | (8) |
| --- | --- | --- | --- | --- | --- | --- | --- | --- |
| Hospital_i_ | 0.012*** | 0.028*** | 0.029*** | 0.028*** | 0.028*** | 0.035*** | 0.036*** | 0.040*** |
|  | [0.004] | [0.005] | [0.005] | [0.005] | [0.005] | [0.005] | [0.005] | [0.005] |
| Students_it_ | -0.010*** | -0.013*** | -0.016*** | -0.013*** | -0.013*** | -0.015*** | -0.014*** | -0.017*** |
|  | [0.003] | [0.003] | [0.003] | [0.003] | [0.003] | [0.003] | [0.003] | [0.003] |
| Students_it_^2^ | 0.001*** | 0.002*** | 0.002*** | 0.002*** | 0.002*** | 0.002*** | 0.002*** | 0.002*** |
|  | [0.000] | [0.000] | [0.000] | [0.000] | [0.000] | [0.000] | [0.000] | [0.000] |
| YearFound_i_ | 0.077*** | 0.080*** | 0.076*** | 0.083*** | 0.087*** | 0.082*** | 0.083*** | 0.071*** |
|  | [0.011] | [0.012] | [0.011] | [0.012] | [0.012] | [0.011] | [0.011] | [0.011] |
| YearFound_i_ ^2^ | -0.002*** | -0.003*** | -0.002*** | -0.003*** | -0.003*** | -0.003*** | -0.003*** | -0.002*** |
|  | [0.000] | [0.000] | [0.000] | [0.000] | [0.000] | [0.000] | [0.000] | [0.000] |
| Private_i_ | -0.009 | -0.019 | -0.011 | -0.028** | -0.035** | -0.019 | -0.021* | -0.036** |
|  | [0.008] | [0.013] | [0.013] | [0.014] | [0.014] | [0.013] | [0.013] | [0.014] |
| Publ_Acad_it_ | 0.009* | 0.009* | 0.006 | 0.010** | 0.011** | 0.011** | 0.012** | 0.010* |
|  | [0.005] | [0.005] | [0.005] | [0.005] | [0.005] | [0.005] | [0.005] | [0.005] |
| Non_personal_it_ |  | 0.008 | 0.808*** |  |  |  |  | 0.942*** |
|  |  | [0.035] | [0.125] |  |  |  |  | [0.134] |
| Non_personal_it_^2^ |  |  | -1.043*** |  |  |  |  | -1.269*** |
|  |  |  | [0.168] |  |  |  |  | [0.183] |
| Core budget_it_ |  |  |  | -0.018 | -0.106** |  |  | -0.093* |
|  |  |  |  | [0.014] | [0.043] |  |  | [0.047] |
| Core budget_it_^2^ |  |  |  |  | 0.087** |  |  | 0.047 |
|  |  |  |  |  | [0.039] |  |  | [0.047] |
| Third party_it_ |  |  |  |  |  | -0.047** | -0.115*** | -0.168*** |
|  |  |  |  |  |  | [0.019] | [0.036] | [0.038] |
| Third party_it_^2^ |  |  |  |  |  |  | 0.109* | 0.152*** |
|  |  |  |  |  |  |  | [0.058] | [0.056] |
| N | 4039 | 2742 | 2742 | 2902 | 2902 | 2710 | 2710 | 2570 |
| R^2^ | 0.47 | 0.44 | 0.45 | 0.44 | 0.44 | 0.45 | 0.45 | 0.48 |

Notes: * p<0.10, ** p<0.05, *** p<0.01, Country and time fixed effects included (not reported). Robust standard errors. Specifications (2)- (5): no data on BG, ES, GR, HR, IS, specification (6)-(8): additionally, no data on CZ.

Hospital: 1-university hospital, 0 –otherwise; university hospital defined as a hospital that has a close link with the HEI, based on the following criteria: a) shared people (i.e. medical doctors also appointed as HEI staff/professors), b) shared facilities and research activities, c) involvement of the hospital in medical education. University hospitals are not necessarily legally part of the HEI, but might be independent organizations with close link with the HEI.

Source: own elaboration based on data from ETER

**Table S6. Determinants of *Non_acad* (additional RHS variable: EU_FP participation), European sample**

|  | (1) | (2) | (3) | (4) | (5) | (6) | (7) | (8) |
| --- | --- | --- | --- | --- | --- | --- | --- | --- |
| EU_FP_it_ | 0.020*** | 0.004 | 0.001 | 0.004 | 0.007 | 0.008 | 0.011* | 0.013** |
|  | [0.006] | [0.005] | [0.005] | [0.005] | [0.005] | [0.006] | [0.006] | [0.006] |
| EU_FP_it_^2^ | -0.008*** | -0.005*** | -0.004*** | -0.005*** | -0.006*** | -0.006*** | -0.007*** | -0.007*** |
|  | [0.001] | [0.001] | [0.001] | [0.001] | [0.001] | [0.001] | [0.001] | [0.001] |
| Students_it_ | -0.009*** | -0.005* | -0.007** | -0.006** | -0.006** | -0.006** | -0.006** | -0.008*** |
|  | [0.003] | [0.003] | [0.003] | [0.003] | [0.003] | [0.003] | [0.003] | [0.003] |
| Students_it_^2^ | 0.001*** | 0.001*** | 0.001*** | 0.001*** | 0.001*** | 0.001*** | 0.001*** | 0.001*** |
|  | [0.000] | [0.000] | [0.000] | [0.000] | [0.000] | [0.000] | [0.000] | [0.000] |
| YearFound_i_ | 0.057*** | 0.054*** | 0.051*** | 0.055*** | 0.057*** | 0.054*** | 0.053*** | 0.044*** |
|  | [0.011] | [0.012] | [0.011] | [0.011] | [0.011] | [0.012] | [0.012] | [0.012] |
| YearFound_i_ ^2^ | -0.002*** | -0.002*** | -0.002*** | -0.002*** | -0.002*** | -0.002*** | -0.002*** | -0.002*** |
|  | [0.000] | [0.000] | [0.000] | [0.000] | [0.000] | [0.000] | [0.000] | [0.000] |
| Private_i_ | -0.007 | -0.015 | -0.007 | -0.026* | -0.034** | -0.013 | -0.015 | -0.027* |
|  | [0.008] | [0.013] | [0.013] | [0.014] | [0.014] | [0.012] | [0.013] | [0.014] |
| Publ_Acad_it_ | 0.012** | 0.025*** | 0.023*** | 0.026*** | 0.026*** | 0.025*** | 0.026*** | 0.024*** |
|  | [0.005] | [0.004] | [0.004] | [0.004] | [0.004] | [0.004] | [0.004] | [0.004] |
| Non_personal_it_ |  | 0.018 | 0.793*** |  |  |  |  | 0.925*** |
|  |  | [0.035] | [0.128] |  |  |  |  | [0.136] |
| Non_personal_it_^2^ |  |  | -1.011*** |  |  |  |  | -1.228*** |
|  |  |  | [0.173] |  |  |  |  | [0.185] |
| Core budget_it_ |  |  |  | -0.024* | -0.116*** |  |  | -0.121** |
|  |  |  |  | [0.014] | [0.044] |  |  | [0.047] |
| Core budget_it_^2^ |  |  |  |  | 0.092** |  |  | 0.088* |
|  |  |  |  |  | [0.039] |  |  | [0.047] |
| Third party_it_ |  |  |  |  |  | -0.012 | -0.068* | -0.096** |
|  |  |  |  |  |  | [0.020] | [0.039] | [0.040] |
| Third party_it_^2^ |  |  |  |  |  |  | 0.088 | 0.106* |
|  |  |  |  |  |  |  | [0.061] | [0.059] |
| N | 4050 | 2742 | 2742 | 2902 | 2902 | 2710 | 2710 | 2570 |
| R^2^ | 0.47 | 0.44 | 0.45 | 0.43 | 0.44 | 0.44 | 0.45 | 0.47 |

Notes: * p<0.10, ** p<0.05, *** p<0.01, Country and time fixed effects included (not reported). Robust standard errors. Specifications (2)- (5): no data on BG, ES, GR, HR, IS, specification (6)-(8): additionally, no data on CZ. EU-FP: participation in EU-FP projects, source: EUPRO

Source: own elaboration based on data from ETER

**Table S7. Determinants of *Admin_Tot* (additional RHS variable: university hospital), USA sample**

|  | (1) | (2) | (3) | (4) | (5) | (6) | (7) | (8) |
| --- | --- | --- | --- | --- | --- | --- | --- | --- |
| Hospital_i_ | 0.019*** | 0.018*** | 0.018*** | 0.018*** | 0.017*** | 0.021*** | 0.019*** | 0.016*** |
|  | [0.005] | [0.005] | [0.005] | [0.005] | [0.005] | [0.005] | [0.005] | [0.005] |
| Students_it_ | -0.003 | -0.003 | -0.003 | -0.004 | -0.003 | -0.004 | -0.005** | -0.005* |
|  | [0.003] | [0.003] | [0.003] | [0.003] | [0.003] | [0.003] | [0.003] | [0.003] |
| Students_it_^2^ | 0.001** | 0.001** | 0.001** | 0.001** | 0.001** | 0.001** | 0.001*** | 0.001** |
|  | [0.000] | [0.000] | [0.000] | [0.000] | [0.000] | [0.000] | [0.000] | [0.000] |
| YearFound_i_ | 0.288*** | 0.282*** | 0.281*** | 0.283*** | 0.271*** | 0.346*** | 0.366*** | 0.356*** |
|  | [0.096] | [0.096] | [0.096] | [0.095] | [0.095] | [0.099] | [0.096] | [0.094] |
| YearFound_i_ ^2^ | -0.008*** | -0.008*** | -0.008*** | -0.008*** | -0.007*** | -0.009*** | -0.010*** | -0.010*** |
|  | [0.003] | [0.003] | [0.003] | [0.003] | [0.003] | [0.003] | [0.003] | [0.003] |
| Private_i_ | -0.002 | -0.003 | -0.003 | -0.01 | -0.020* | -0.005 | -0.002 | -0.031*** |
|  | [0.004] | [0.004] | [0.004] | [0.007] | [0.010] | [0.004] | [0.004] | [0.010] |
| Publ_Acad_it_ | 0.051*** | 0.052*** | 0.052*** | 0.051*** | 0.050*** | 0.056*** | 0.054*** | 0.053*** |
|  | [0.003] | [0.003] | [0.003] | [0.003] | [0.003] | [0.004] | [0.004] | [0.004] |
| Non_personal_it_ |  | -0.023 | 0.045 |  |  |  |  | -0.303 |
|  |  | [0.032] | [0.336] |  |  |  |  | [0.412] |
| Non_personal_it_^2^ |  |  | -0.064 |  |  |  |  | 0.274 |
|  |  |  | [0.312] |  |  |  |  | [0.390] |
| Core budget_it_ |  |  |  | -0.02 | -0.087* |  |  | -0.141*** |
|  |  |  |  | [0.015] | [0.051] |  |  | [0.051] |
| Core budget_it_^2^ |  |  |  |  | 0.087 |  |  | 0.141** |
|  |  |  |  |  | [0.060] |  |  | [0.061] |
| Third party_it_ |  |  |  |  |  | -0.041** | 0.130*** | 0.144*** |
|  |  |  |  |  |  | [0.020] | [0.045] | [0.046] |
| Third party_it_^2^ |  |  |  |  |  |  | -0.389*** | -0.448*** |
|  |  |  |  |  |  |  | [0.100] | [0.102] |
| N | 2042 | 2042 | 2042 | 2042 | 2042 | 2042 | 2042 | 2042 |
| R^2^ | 0.42 | 0.42 | 0.42 | 0.42 | 0.42 | 0.42 | 0.43 | 0.43 |

Notes: * p<0.10, ** p<0.05, *** p<0.01, State and time fixed effects included (not reported). Robust standard errors.

Hospital: 1 - institution has hospital, 0 – otherwise. Institution is classified as having hospital on the bases of financial data: reported either revenues from sales and services of hospitals or expenses for hospital services.

Source: own elaboration based on data from IPEDS

**Table S 8. Determinants of *Non_acad*, European sample, outliers detected as *Non_acad* lower than 1^st^ percentile or higher than 99^th^ percentile**

|  | (1) | (2) | (3) | (4) | (5) | (6) | (7) | (8) |
| --- | --- | --- | --- | --- | --- | --- | --- | --- |
| Students_it_ | -0.010*** | -0.006** | -0.009*** | -0.007*** | -0.007*** | -0.007** | -0.006** | -0.008*** |
|  | [0.002] | [0.003] | [0.003] | [0.003] | [0.003] | [0.003] | [0.003] | [0.003] |
| Students_it_^2^ | 0.001*** | 0.001*** | 0.002*** | 0.001*** | 0.001*** | 0.001*** | 0.001*** | 0.001*** |
|  | [0.000] | [0.000] | [0.000] | [0.000] | [0.000] | [0.000] | [0.000] | [0.000] |
| YearFound_i_ | 0.078*** | 0.079*** | 0.075*** | 0.082*** | 0.083*** | 0.081*** | 0.081*** | 0.068*** |
|  | [0.011] | [0.012] | [0.012] | [0.012] | [0.012] | [0.012] | [0.012] | [0.012] |
| YearFound_i_ ^2^ | -0.002*** | -0.003*** | -0.002*** | -0.003*** | -0.003*** | -0.003*** | -0.003*** | -0.002*** |
|  | [0.000] | [0.000] | [0.000] | [0.000] | [0.000] | [0.000] | [0.000] | [0.000] |
| Private_i_ | 0.006 | -0.001 | 0.009 | -0.008 | -0.011 | 0.000 | -0.001 | -0.008 |
|  | [0.008] | [0.013] | [0.012] | [0.014] | [0.014] | [0.013] | [0.013] | [0.014] |
| Publ_Acad_it_ | 0.017*** | 0.019*** | 0.016*** | 0.019*** | 0.020*** | 0.023*** | 0.023*** | 0.022*** |
|  | [0.003] | [0.004] | [0.004] | [0.004] | [0.004] | [0.004] | [0.004] | [0.004] |
| Non_personal_it_ |  | 0.007 | 0.834*** |  |  |  |  | 0.945*** |
|  |  | [0.035] | [0.128] |  |  |  |  | [0.137] |
| Non_personal_it_^2^ |  |  | -1.080*** |  |  |  |  | -1.266*** |
|  |  |  | [0.173] |  |  |  |  | [0.187] |
| Core budget_it_ |  |  |  | -0.014 | -0.047 |  |  | -0.037 |
|  |  |  |  | [0.014] | [0.042] |  |  | [0.045] |
| Core budget_it_^2^ |  |  |  |  | 0.033 |  |  | -0.002 |
|  |  |  |  |  | [0.037] |  |  | [0.046] |
| Third party_it_ |  |  |  |  |  | -0.035** | -0.063* | -0.125*** |
|  |  |  |  |  |  | [0.018] | [0.036] | [0.037] |
| Third party_it_^2^ |  |  |  |  |  |  | 0.046 | 0.106* |
|  |  |  |  |  |  |  | [0.059] | [0.057] |
| N | 3948 | 2726 | 2726 | 2886 | 2886 | 2696 | 2696 | 2556 |
| R^2^ | 0.4 | 0.43 | 0.44 | 0.42 | 0.42 | 0.43 | 0.43 | 0.45 |

Notes: * p<0.10, ** p<0.05, *** p<0.01, Country and time fixed effects included (not reported). Robust standard errors. Specifications (2)- (5): no data on BG, ES, GR, HR, IS, specification (6)-(8): additionally, no data on CZ

Source: own elaboration based on data from ETER

**Table S 9. Determinants of *Non_acad*, European sample, outliers detected as values of *Non_acad* three standard deviations away from the mean**

|  | (1) | (2) | (3) | (4) | (5) | (6) | (7) | (8) |
| --- | --- | --- | --- | --- | --- | --- | --- | --- |
| Students_it_ | -0.009*** | -0.009*** | -0.012*** | -0.010*** | -0.009*** | -0.010*** | -0.009*** | -0.011*** |
|  | [0.003] | [0.003] | [0.003] | [0.003] | [0.003] | [0.003] | [0.003] | [0.003] |
| Students_it_^2^ | 0.001*** | 0.002*** | 0.002*** | 0.002*** | 0.002*** | 0.002*** | 0.002*** | 0.002*** |
|  | [0.000] | [0.000] | [0.000] | [0.000] | [0.000] | [0.000] | [0.000] | [0.000] |
| YearFound_i_ | 0.075*** | 0.075*** | 0.071*** | 0.077*** | 0.081*** | 0.076*** | 0.077*** | 0.067*** |
|  | [0.011] | [0.012] | [0.012] | [0.012] | [0.012] | [0.012] | [0.012] | [0.012] |
| YearFound_i_ ^2^ | -0.002*** | -0.002*** | -0.002*** | -0.002*** | -0.003*** | -0.002*** | -0.002*** | -0.002*** |
|  | [0.000] | [0.000] | [0.000] | [0.000] | [0.000] | [0.000] | [0.000] | [0.000] |
| Private_i_ | -0.01 | -0.021 | -0.013 | -0.031** | -0.040*** | -0.018 | -0.02 | -0.041*** |
|  | [0.008] | [0.013] | [0.013] | [0.014] | [0.014] | [0.013] | [0.013] | [0.015] |
| Publ_Acad_it_ | 0.014*** | 0.022*** | 0.019*** | 0.023*** | 0.024*** | 0.027*** | 0.029*** | 0.027*** |
|  | [0.004] | [0.004] | [0.004] | [0.004] | [0.004] | [0.004] | [0.004] | [0.004] |
| Non_personal_it_ |  | 0.022 | 0.799*** |  |  |  |  | 0.926*** |
|  |  | [0.035] | [0.128] |  |  |  |  | [0.139] |
| Non_personal_it_^2^ |  |  | -1.012*** |  |  |  |  | -1.225*** |
|  |  |  | [0.173] |  |  |  |  | [0.189] |
| Core budget_it_ |  |  |  | -0.024* | -0.131*** |  |  | -0.122** |
|  |  |  |  | [0.014] | [0.045] |  |  | [0.048] |
| Core budget_it_^2^ |  |  |  |  | 0.106*** |  |  | 0.07 |
|  |  |  |  |  | [0.040] |  |  | [0.048] |
| Third party_it_ |  |  |  |  |  | -0.046** | -0.105*** | -0.141*** |
|  |  |  |  |  |  | [0.019] | [0.038] | [0.039] |
| Third party_it_^2^ |  |  |  |  |  |  | 0.095 | 0.113** |
|  |  |  |  |  |  |  | [0.061] | [0.057] |
| N | 4062 | 2748 | 2748 | 2908 | 2908 | 2716 | 2716 | 2576 |
| R^2^ | 0.47 | 0.43 | 0.44 | 0.42 | 0.43 | 0.44 | 0.44 | 0.46 |

Notes: * p<0.10, ** p<0.05, *** p<0.01, Country and time fixed effects included (not reported). Robust standard errors. Specifications (2)- (5): no data on BG, ES, GR, HR, IS, specification (6)-(8): additionally, no data on CZ

Source: own elaboration based on data from ETER

**Table S 10. Determinants of *Non_acad*, European sample, different specifications considering *Third party*_it_**

|  | (1) | (2) | (3) | (4) | (5) | (6) |
| --- | --- | --- | --- | --- | --- | --- |
| Students_it_ | -0.009*** | -0.008*** | -0.008*** | -0.008*** | -0.008*** | -0.008*** |
|  | [0.003] | [0.003] | [0.003] | [0.003] | [0.003] | [0.003] |
| Students_it_^2^ | 0.001*** | 0.001*** | 0.001*** | 0.001*** | 0.001*** | 0.001*** |
|  | [0.000] | [0.000] | [0.000] | [0.000] | [0.000] | [0.000] |
| YearFound_i_ | 0.068*** | 0.067*** | 0.064*** | 0.063*** | 0.068*** | 0.067*** |
|  | [0.012] | [0.012] | [0.012] | [0.012] | [0.012] | [0.012] |
| YearFound_i_ ^2^ | -0.002*** | -0.002*** | -0.002*** | -0.002*** | -0.002*** | -0.002*** |
|  | [0.000] | [0.000] | [0.000] | [0.000] | [0.000] | [0.000] |
| Private_i_ | -0.029** | -0.028** | -0.02 | -0.02 | -0.028* | -0.026* |
|  | [0.014] | [0.014] | [0.015] | [0.015] | [0.014] | [0.015] |
| Publ_Acad_it_ | 0.023*** | 0.024*** | 0.027*** | 0.027*** | 0.023*** | 0.024*** |
|  | [0.004] | [0.004] | [0.004] | [0.004] | [0.004] | [0.004] |
| Non_personal_it_ | 0.913*** | 0.922*** | 0.875*** | 0.880*** | 0.913*** | 0.922*** |
|  | [0.135] | [0.136] | [0.139] | [0.140] | [0.135] | [0.136] |
| Non_personal_it_^2^ | -1.234*** | -1.243*** | -1.164*** | -1.169*** | -1.233*** | -1.245*** |
|  | [0.184] | [0.184] | [0.190] | [0.192] | [0.184] | [0.185] |
| Core budget_it_ | -0.125*** | -0.100** | -0.101** | -0.095** | -0.120** | -0.096** |
|  | [0.046] | [0.047] | [0.048] | [0.048] | [0.047] | [0.047] |
| Core budget_it_^2^ | 0.083* | 0.06 | 0.076 | 0.072 | 0.079* | 0.058 |
|  | [0.046] | [0.047] | [0.048] | [0.048] | [0.047] | [0.047] |
| Third party_it_ | **-0.055***** | **-0.111***** | **-0.065***** | **-0.098*** | **-0.057***** | **-0.117***** |
|  | [0.018] | [0.037] | [0.021] | [0.053] | [0.020] | [0.043] |
| Third party_it_^2^ |  | 0.088 |  | **0.071** |  | **0.103** |
|  |  | [0.057] |  | [0.114] |  | [0.075] |
| N | **2570** | **2570** | **2543** | **2543** | **2566** | **2566** |
| R^2^ | 0.46 | 0.46 | 0.47 | 0.47 | 0.46 | 0.46 |

Notes: * p<0.10, ** p<0.05, *** p<0.01, Country and time fixed effects included (not reported). Robust standard errors. Specifications 1 and 2 full sample, specifications 2 - 6 observations with Third party_it_ outliers excluded: specifications 3 and 4: outliers defined as values of Third party_it_ higher than 99 percentile, specifications: 5 and 6 outliers with values of Third party_it_ higher than 90%.

Source: own elaboration based on data from ETER

**Table S 11. Determinants of *Non_acad*, European (specifications 1 and 2) and US (specifications 3 and 4) samples, estimations based on mean-centred continuous variables**

|  | Europe | | USA | |
| --- | --- | --- | --- | --- |
|  | (1) | (2) | (3) | (4) |
| cStudents_it_ | -0.001 | -0.001 | -0.001 | -0.002 |
|  | [0.002] | [0.002] | [0.001] | [0.001] |
| cStudents_it_^2^ | 0.001* | 0.001* | 0.001*** | 0.002*** |
|  | [0.000] | [0.000] | [0.000] | [0.000] |
| YearFound_i_ | 0.067*** | 0.066*** | 0.341*** | 0.364*** |
|  | [0.012] | [0.012] | [0.100] | [0.099] |
| YearFound_i_ ^2^ | -0.002*** | -0.002*** | -0.009*** | -0.010*** |
|  | [0.000] | [0.000] | [0.003] | [0.003] |
| Private_i_ | -0.080*** | -0.081*** | -0.032*** | -0.032*** |
|  | [0.019] | [0.020] | [0.008] | [0.008] |
| cPubl_Acad_it_ | 0.024*** | 0.023*** | 0.056*** | 0.056*** |
|  | [0.004] | [0.004] | [0.004] | [0.004] |
| cNon_personal_it_ | 0.122*** | 0.121*** | -0.044 | -0.042 |
|  | [0.033] | [0.034] | [0.033] | [0.033] |
| cNon_personal_it_^2^ | -1.327*** | -1.326*** | 0.382 | 0.569 |
|  | [0.330] | [0.329] | [0.337] | [0.392] |
| cCore budget_it_ | -0.023 | -0.022 | -0.063*** | -0.066*** |
|  | [0.014] | [0.014] | [0.017] | [0.018] |
| cCore budget_it_^2^ | 0.290*** | 0.301*** | 0.137*** | 0.139*** |
|  | [0.055] | [0.059] | [0.047] | [0.047] |
| cThird party_it_ | -0.081*** | -0.072*** | -0.035* | -0.001 |
|  | [0.017] | [0.021] | [0.021] | [0.022] |
| cThird party_it_^2^ |  | -0.036 |  | -0.294*** |
|  |  | [0.058] |  | [0.097] |
| N | 2,570 | 2,570 | 2,042 | 2,042 |
| R^2^ | 0.47 | 0.47 | 0.42 | 0.43 |

Notes: * p<0.10, ** p<0.05, *** p<0.01, Country (European sample)/ state (USA sample) and time fixed effects included (not reported). Robust standard errors. Europe: specifications 1 and 2, USA: specifications 3 and 4 Mean-centred continuous variables related to the country (in case of Europe) and state (in case of the USA) mean.
